# Off-label and investigational drugs in the treatment of alcohol use disorder: A critical review

**DOI:** 10.3389/fphar.2022.927703

**Published:** 2022-10-03

**Authors:** Pascal Valentin Fischler, Michael Soyka, Erich Seifritz, Jochen Mutschler

**Affiliations:** ^1^ Department for Gynecology and Obstetrics, Women’s Clinic Lucerne, Cantonal Hospital of Lucerne, Lucerne, Switzerland; ^2^ Psychiatric Hospital University of Munich, Munich, Germany; ^3^ Director of the Clinic for Psychiatry, Psychotherapy and Psychosomatics, Psychiatric University Clinic Zürich, Zürich, Switzerland; ^4^ Meiringen Private Clinic, Meiringen, Switzerland

**Keywords:** alcohol use disorder, pharmacological treatment, off-label, relapse prophylaxis, baclofen, topiramate, ondansetron, varenicline

## Abstract

Compounds known to be successful in the treatment of alcohol use disorder include the aversive agent, Disulfiram, the glutamatergic NMDA receptor antagonist, Acamprosate, and the opioid receptor antagonists, Naltrexone and Nalmefene. Although all four are effective in maintaining abstinence or reduction of alcohol consumption, only a small percentage of patients receive pharmacological treatment. In addition, many other medications have been investigated for their therapeutic potential in the treatment of alcohol use disorder. In this review we summarize and compare Baclofen, Gabapentin, Topiramate, Ondansetron, Varenicline, Aripiprazole, Quetiapine, Clozapine, Antidepressants, Lithium, Neuropeptide Y, Neuropeptide S, Corticotropin-releasing factor antagonists, Oxytocin, PF-05190457, Memantine, Ifenprodil, Samidorphan, Ondelopran, ABT-436, SSR149415, Mifepristone, Ibudilast, Citicoline, Rimonabant, Surinabant, AM4113 and Gamma-hydroxybutyrate While some have shown promising results in the treatment of alcohol use disorder, others have disappointed and should be excluded from further investigation. Here we discuss the most promising results and highlight medications that deserve further preclinical or clinical study. Effective, patient-tailored treatment will require greater understanding provided by many more preclinical and clinical studies.

## 1 Introduction

Alcohol use disorder imposes a large medical risk on affected patients and represents an enormous medical and economic burden for society, as well as being the world’s most prevalent substance use disorder (age-standardized worldwide prevalence 1,321 cases per 100,000 people) (GBD 2016; Alcohol and Drug Use Collaborators, 2018). The lifetime risk for development of alcohol use disorder (AUD) is estimated at 5.1%–8.6%. Alcohol-associated liver disease is globally the major cause of liver-related morbidity and mortality (Asrani et al., 2021), and alcohol use disorder is the most common substance use-related reason for emergency department presentation (Xierali et al., 2021). Furthermore, there is a potentiation of harm due to frequent co-occurrence of alcohol disorder with depression and other comorbid psychiatric disorders (McHugh and Weiss, 2019), in addition to the association with other substance use disorders (Alcover et al., 2021). The pathophysiology of alcohol use disorder is still not completely understood due to the enormous complexity of neuronal network system and multigenetic influences. Many different neurotransmitter systems are involved in the pathophysiological genesis and maintenance of alcohol use disorder, such as the mesolimbic dopamine system, GABAergic neurotransmitter system, serotoninergic-, acetyl cholinergic-, adrenergic- and NMDA-receptor systems, as well as the endogenous opioid receptors and the endocannabinoid system. As well as neuroendocrine hormones and the hypothalamic-pituitary-adrenal axis of central stress system, intraneuronal pre- and postsynaptic regulation systems also seem to play a role. Therefore, many different pharmacological target points have been investigated in relation to the treatment of alcohol use disorders (Spanagel, 2009). As the epidemiology, symptomatology, pathophysiology and diagnosis of alcohol use disorder are all well described, we only refer to the relevant literature and national guidelines (Spanagel, 2009; Sommer and Spanagel, 2013; Günthner et al., 2018). Data suggests that alcohol use disorder is still heavily underreported and that many cases go undetected. Amongst diagnosed cases, only a small proportion receives adequate psychotherapeutic treatment and an even smaller percentage is allocated to pharmacological treatment and anti-craving medication (Wallhed Finn et al., 2021; Larsen et al., 2022). Despite the fact that some medications are well-proven and many new substances are currently under investigation, pharmacologic treatment of alcohol use disorder remains underutilized and undervalued (Cohen et al., 2022).

### 1.1 Current pharmacological treatment of alcohol use disorder

Besides psychotherapeutic treatment methods for relapse prophylaxis in patients with alcohol use disorder, which have proven effective in supporting abstinence and preventing relapse (Miller and Wilbourne, 2002), there are four established options for the pharmacological treatment of alcohol use disorder. These four medications, the aversive agent disulfiram, the glutamatergic NMDA receptor antagonist acamprosate, and the opioid receptor antagonists naltrexone and nalmefene, are currently approved for the treatment of alcohol use disorders (Soyka and Lieb, 2015). Disulfiram is an aversive drug causing nausea and vomiting in case of alcohol consumption due to its irreversible inhibition of hepatic aldehyde dehydrogenases and production of toxic acetaldehyde. It is therefore used to maintain strict abstinence and should prevent relapse (Mutschler et al., 2016). The other two medications for which the main therapeutic goal is to maintain abstinence due to reduction of alcohol-craving are the NMDA receptor antagonist acamprosate (Kiefer and Mann, 2010), and the long-acting opioid receptor antagonist naltrexone (Kirchoff et al., 2021). Various meta-analyses and reviews have described the effectiveness of these medications in treating alcohol use disorder (Garbutt et al., 1999; Kenna et al., 2004a, 2004b; Soyka and Roesner, 2006; Jonas et al., 2014; Soyka and Mutschler, 2016). Despite convincing data, only 30% of patients with alcohol use disorder (AUD) receive any treatment and less than 10% are treated with pharmacological anti-craving medications for alcohol consumption relapse prophylaxis (Hasin et al., 2007; Jonas et al., 2014).

### 1.2 The search for additional anti-craving drugs

Even though established pharmacological treatments of alcohol use disorder have been shown to be effective in relapse prophylaxis, these medications have not yet found widespread success and acceptance in affected patient populations, mainly due to treatment-related side effects. Therefore, the search for new pharmacological substances for treatment of alcohol use disorder should be encouraged (Shen, 2018a). As shown in the case of Baclofen, the promotion of this anti-craving drug by Olivier Ameisen attracted public attention regarding the inadequate pharmacological treatment options provided by currently approved drugs for the treatment of alcohol use disorder (Ameisen, 2011). Therefore, studies of off-label anti-craving medications are justified. When seeking new pharmacological substances to treat alcohol use disorders, we should take into account the characteristics of an optimal anti-craving drug as proposed by M. Soyka. These include a low relapse potential, no psychotropic effect or dependence potential of the drug itself, no interaction with alcohol, no hepatotoxic effects, few side effects, lack of an unfavorable side-effect profile, and suitability in patients with a reduced general health. Understandably these criteria cannot all be met, but any new pharmacological treatment should be evaluated in light of these criteria. In order to find new anti-craving drugs more research is needed to understand pathogenesis of alcohol use disorder (Lundberg et al., 2021), and an animal model is now available that can be used for screening potential treatments for alcohol use disorder (Bell et al., 2017).

## 2 Methods of literature research

The objective of this paper is to provide an overview of new developments in the pharmacological treatment of alcohol use disorders, with a thematic focus on off-label medications and pharmacogenetic applications. This paper is intended to broaden clinician and researcher horizons regarding medication classes not currently in use in clinical practice. Some show promising initial results and may become future clinical standards in first- or second-line pharmacotherapy of alcohol use disorders. This review paper mainly concerns the clinical research setting and as such will help evaluate the benefit of the further research effort on specific off-label medications. Equally, it may also provide input for preliminary preclinical studies searching for new anti-craving drugs that could be tested in an animal model of alcohol use disorder.

This paper is based on systematic review, literature research and data extraction from the PubMed database concerning current therapeutic standards and existing publications on the topic of off-label medications. In addition, ClinicalTrials.gov was analyzed for recent developments, new pharmacological substances and upcoming research publications. We also considered several national clinical guidelines and meta-analyses concerning various specific anti-craving drugs.

The following key words were used in literature research of PubMed: alcohol, alcohol use disorder, alcohol dependence, alcohol consumption, relapse prophylaxis, anti-craving, pharmacological treatment and off-label use. These terms were used in combination with the different medications discussed in this review. Approximately 1,200 papers were found and reviewed.

## 3 Off-label medication for relapse prophylaxis

A variety of different medications with a diverse range of actions have been preclinically or clinically assessed in the treatment of alcohol use disorder (Hartwell et al., 2022). Some gave promising results, while others were ineffective or produced inconclusive results. In this overview we review and discuss the latest data on the various medications in order to prioritize ongoing or future clinical investigations.

### 3.1 Baclofen

Baclofen is a selective agonist of the B-subunit of the metabotropic GABA receptor (GABA-B) and is approved as a muscle relaxant in the treatment of neurological spasticity in patients with spinal cord lesions or multiple sclerosis (Dario and Tomei, 2004; Bowery, 2006). Due to the active promotion of baclofen as an anti-craving drug by the cardiologist Olivier Ameisen, who used high-dose baclofen to treat his personal alcohol use disorder, this medication has received a lot of public interest (Ameisen, 2005). The therapeutic effect of baclofen in patients with alcohol use disorder is not completely understood, but likely involves two pathways. Baclofen impacts the regulation of emotional behavior through activation of GABA-B receptors in the limbic system, which leads to improved control of anxiety, a frequent comorbidity of alcohol use disorder (Addolorato et al., 2009; Holtyn and Weerts, 2022). Due to the ability of GABA-B receptor agonists and GABA-B receptor allosteric modulators to downregulate the neuronal stress circuit, these medications have been investigated for therapeutic use in stress-related psychiatric disorders including anxiety, mood-disturbances and alcohol use disorder (Morley et al., 2014; Felice et al., 2022). In addition, through the local suppression of dopaminergic neurons baclofen reduces the positive reinforcement of alcohol, which is mediated through trigger-stimulated dopamine release in the limbic system (Addolorato and Leggio, 2010).

Upon oral administration baclofen is rapidly and almost entirely absorbed via the gastrointestinal tract and reaches plasma peak concentrations after 2–3 h (El-Husseini et al., 2011). Due to a relatively short half-life of 2–6 h and its unaltered elimination via the kidneys, the drug has to be taken 3–4 times daily (Soyka and Lieb, 2015). Additional pharmacokinetic studies found a linear relationship between administered oral dose and plasma concentration, even at doses of over 120 mg/day (Chevillard et al., 2017). A very high inter-individual variability could not be explained by demographic factors. In view of its unaltered renal excretion, baclofen has potential applications even in patients with compromised hepatic function (Addolorato et al., 2007; El-Husseini et al., 2011; Chevillard et al., 2017).

Although baclofen received temporary approval by the French drug agency (ANSM) in 2014 for the new indication of alcohol use disorder, conflicting study results have led to scientific controversy (Soyka et al., 2017). Results from preclinical and small preliminary clinical trials have shown that baclofen is effective in reducing alcohol intake and craving in patients with alcohol use disorder (Addolorato et al., 2000; Colombo et al., 2000). These early results were reproduced in several randomized clinical trials conducted in Italy using baclofen doses of 10–30 mg/day (Addolorato et al., 2000, 2002, 2007, 2009, 2011; Addolorato and Leggio, 2010). In contrast to these results, a double-blind, placebo-controlled, randomized clinical study conducted in the US failed to find evidence that baclofen (30 mg/day) is superior to placebo in the treatment of alcohol use disorder (Garbutt et al., 2010). These differences may be attributable to methodological variation such as study design, male/female ratio, treatment duration, intensity of psychological support and patient characteristics. The Italian studies appear to have included more patients with severe alcohol use disorder (Addolorato and Leggio, 2010). Nevertheless, other studies have also reported negative results (Ponizovsky et al., 2015; Hauser et al., 2017). A small double-blind, randomized, placebo-controlled clinical trial with 50 mg/day (Krupitsky et al., 2015) and a double-blind, randomized, 3-arm clinical trial comparing baclofen 30 mg/day and 60 mg/day against placebo also failed to show that baclofen was effective in reducing alcohol intake in alcohol use disorder (Morley et al., 2014).

Interestingly, a secondary analysis of this 3-arm clinical trial, with stratification for anxiety comorbidity, showed a significant reduction of relapse risk in patients with alcohol use disorder and comorbid anxiety disorder (Morley et al., 2014). These secondary findings partially agree with the results of the Italian studies, where baclofen was not only effective in reducing alcohol intake and craving, but also reduced symptoms of anxiety and stress. Baclofen is thought to interact with the stress circuit in the brain and other neuroendocrine systems, resulting in the regulation of stress and emotional behavior (Addolorato et al., 2009).

A retrospective study of patients with alcohol use disorder found that baclofen reduced alcohol craving, even though no decrease in alcohol consumption was seen. The most interesting finding of this study was the identification of two subpopulations that differ in their response time to baclofen treatment. While the early-responders show a medication effect within days, late-responders require baclofen treatment for several weeks before an anti-craving effect becomes evident (Imbert et al., 2015). However, it was not possible to predict from patient data which subpopulation a patient might belong to. This phenomenon could be one explanation for inconsistent study results between the various clinical trials, besides the widely varying study designs and medication dosages.

Due to the extremely high baclofen dose (up to 270 mg/d) used by Olivier Ameisen in his experimental self-treatment (Ameisen, 2005), and the inconsistent study data produced in previous clinical trials with low or intermediate baclofen concentrations, researchers have recently performed studies with high dosages (Müller et al., 2015; Beraha et al., 2016; Reynaud et al., 2017). This tendency towards higher dosages also relates to the suspected dose-response effect of baclofen described in previous studies (Addolorato et al., 2011). However, a randomized, placebo-controlled study of 320 patients with AUD, attending a treatment program with a target dose of 180 mg/d during a 26-week clinical trial and a follow-up of 20 weeks, did not find a significantly higher abstinence rate in the baclofen group compared to the placebo control. Nevertheless, a tendential reduction of alcohol consumption and a significant anti-craving effect of baclofen could be demonstrated (Reynaud et al., 2017). Even more disappointing results were reported in a multicenter, double-blind placebo-controlled clinical trial with baclofen doses of 30 mg/d and 130 mg/d. Neither the low-dose nor the high-dose group showed a significant effect of treatment compared to placebo. Furthermore, frequent drug side effects, especially in the high-dose group, were reported (Beraha et al., 2016). In complete contrast to the findings above, a randomized double-blind, placebo-controlled clinical trial at the Berlin Charité, including 56 patients with individual titration of baclofen up to 270 mg/d, reported significantly higher total abstinence and abstinence duration in patients treated with baclofen. Perhaps due to the individual titration of baclofen, no serious adverse treatment effects were reported (Müller et al., 2015).

Nevertheless, this trend towards the administration of high-dose baclofen leads to important safety concerns (Akosile and Klan, 2016; Boels et al., 2017), and serious self-poisoning due to baclofen treatment in AUD is being increasingly reported (Léger et al., 2017; Tyson et al., 2022). A serious safety risk is the occurrence of major sedation, especially in high baclofen doses up to 300 mg/d. The sedation risk is directly related to the dose of baclofen and the amount of consumed alcohol. This is especially concerning in patients without successful abstinence, as alcohol consumption increases during relapse (Rolland et al., 2015). One report even described baclofen overdose as mimicking brain death in a case of deep coma (Sullivan et al., 2012). Besides secondary effects, baclofen use also poses the problem of withdrawal symptoms, including delirium and seizure in the event of rapid discontinuation (Leung et al., 2006; Franchitto et al., 2014; Kapil et al., 2014).

Despite these safety concerns, baclofen has a relatively high acceptance among patients with AUD, suggesting that baclofen could potentially become an accepted alternative strategy for patients with treatment-refractory alcohol use disorder, especially in heavy-drinkers (Pierce et al., 2018; Leggio and Litten, 2021). To ensure an efficient and safe administration more clinical studies are needed to specify indications and patient selection, as well as to refine individual titration of baclofen dose (Morley et al., 2018; Rose and Jones, 2018; Garbutt et al., 2021). Even though data on baclofen are conflicting, this medication appears to have the potential to become a valid alternative treatment, especially in patients with severely reduced liver function or comorbid anxiety disorder (de Beaurepaire et al., 2018).

### 3.2 Anticonvulsants

#### 3.2.1 Gabapentin

Gabapentin belongs to the pharmacological group of anticonvulsants and can be used for treatment of epileptic diseases and neuropathic pain due to its inhibition of presynaptic voltage-gated Na+ and Ca2+ cannels in neuronal cells (Rogawski and Löscher, 2004; Dickenson and Ghandehari, 2007; Landmark, 2007). Furthermore, gabapentin affects the regulation of neurotransmitter release, preventing the release of neurotransmitters such as glutamate (Bonnet et al., 1999; Coderre et al., 2007; Prisciandaro et al., 2021). In a rat model, gabapentin led to the inhibition of K + -triggered glutamate release in the neocortical and hippocampal brain area (Dooley et al., 2000; Cunningham et al., 2004). Gabapentin is also effective in treating somatic symptoms during alcohol withdrawal (Voris et al., 2003; Mariani et al., 2006), as well as in reducing withdrawal-induced CNS hyperexcitability (Watson et al., 1997). The same effects can be seen not only in alcohol withdrawal, but also in the symptomatic treatment of opiate withdrawal (Martí;nez-Raga et al., 2004).

Comparing gabapentin to the established therapeutic lorazepam in the treatment of alcohol withdrawal, a double-blind clinical trial showed that gabapentin was statistically superior but clinically similar to lorazepam (Myrick et al., 2009).

While gabapentin has shown promising results regarding symptomatic treatment, craving and anxiety reduction during alcohol withdrawal, further studies on gabapentin dosage are needed due to insufficient titration data (Berlin et al., 2015; Leung et al., 2015). With regard to relapse prophylaxis of alcohol use disorder, gabapentin significantly reduced heavy drinking in a number of clinical trials, but failed to show any difference from placebo as concerns craving reduction or preservation of abstinence (Pani et al., 2014). The same results were reproduced in a recent meta-analysis where gabapentin was effective in reducing the percentage of heavy drinking days, but did not change any other measurement endpoint compared to placebo (Kranzler et al., 2019).

Contrasting findings were reported by a randomized, double-blind, placebo-controlled clinical trial. This 12-week, three-arm trial included 150 participants, with oral gabapentin administration of 0 mg (placebo), 900 mg/d or 1800 mg/d, and noted both a significant reduction of heavy drinking, mood stabilization and improvement of sleep quality, together with a significant reduction of craving. However, the most important result was perhaps the significant increase in abstinence rate in patients undergoing gabapentin treatment. The abstinence rate showed a dose-dependent increase from 4.1% for placebo to 11.1% abstinence in the 900 mg/d group and 17.0% in the 1800 mg/d group. Even though there were no reports of serious side effects or drug-related adverse events, at 57% (85 of 150 participants) the study completion rate was very low. This low completion rate likely caused a bias in study results, despite similarities in completion across the three groups (Mason et al., 2014).

Due to the dose-dependent effect of gabapentin, as shown in this study, there are safety concerns regarding overdose and abuse (Mersfelder and Nichols, 2016; Smith et al., 2016; Haukka et al., 2018). In a study of intentional drug overdose based on data from a national self-harm registry, a strong increase in emergency department presentation was found for gabapentin overdose. While only 0.5% of intentional drug overdoses in 2007 involved gabapentin, this increased to 5.5% by 2015. Over one third (37.2%) of patients were found to be co-intoxicated with alcohol (Daly et al., 2017). On the other hand, in a study of gabapentin safety in the treatment of substance use disorders, gabapentin was not found to be especially harmful or lethal compared to other prescribed psychotropic drugs (Howland, 2014). Therefore, gabapentin treatment can be considered safe based on rare drug-related adverse events (Mason et al., 2018).

Even though gabapentin can significantly reduce heavy drinking, data concerning craving and abstinence are conflicting (Ahmed et al., 2019; Kranzler et al., 2019). Therefore, new therapies based on different gabapentin dosages or formulas, as well as possible combination therapies with established anti-craving medications, are still under investigation. In a randomized, double-blind, placebo-controlled multisite clinical trial of Gabapentin Enacarbil Extended-Release (GE-XR), a gabapentin-prodrug with intracorporal enzymatic activation into bio-active gabapentin, GE-XR 600 mg twice a day was not found to be effective in reducing alcohol consumption or craving compared to placebo. Furthermore, no beneficial clinical effect could be found for other drinking-behavior measurements, sleep problems, smoking, depression or anxiety symptoms. Therefore GE-XR does not reduce alcohol consumption or craving in patients with alcohol use disorder (Falk et al., 2019b).

In a randomized, double-blind, placebo-controlled, three-arm clinical trial on co-medication with naltrexone and gabapentin, 150 patients with alcohol use disorder were randomized to either double-placebo, naltrexone-only (50 mg/d) or to combined naltrexone (50 mg/d)–gabapentin (up to 1200 mg/d) co-therapy. The addition of gabapentin to the naltrexone treatment protocol resulted in a significant improvement in heavy drinking compared to the naltrexone alone (Anton et al., 2011).

Overall, data on gabapentin use in the treatment of alcohol use disorder are conflicting. Even though gabapentin has shown good results concerning alcohol withdrawal (Anton et al., 2020), but does not seem to be effective as a first line therapy for relapse prophylaxis, and while it can reduce heavy drinking behavior, it does not increase abstinence. In summary, use of gabapentin as an adjunct to established anti-craving medications such as naltrexone seems more promising than single-therapy use of gabapentin.

#### 3.2.2 Topiramate

Topiramate is an antiepileptic drug, approved for the treatment of migraine and epilepsy (French et al., 2004; Wenzel et al., 2006), which promotes GABAergic inhibition of its non-benzodiazepine receptor and reduces glutamate excitatory action at kainate receptors and the AMPA receptor (alpha-amino-3 hydroxy-5 metylisoxazole-4 propionic receptor) (White et al., 2000; Angehagen et al., 2005). As regards pharmacokinetic and pharmacodynamic properties, topiramate has 80% bioavailability and maximal plasma drug concentrations are reached 1.3–1.7 h after oral administration. Exhibiting low plasma-protein binding (15%) and with a half-life of 19–23 h, with repetitive drug ingestion a steady state plasma concentration is reached after approximately 4 days. Around 80% of topiramate is renally excreted in an unchanged state, while approximately 20% undergoes metabolic inactivation (Perucca and Bialer, 1996; Garnett, 2000; Shank et al., 2000; Johnson and Ait-Daoud, 2010).

Besides its use in epilepsy and migraine, topiramate has been investigated in relation to other medical conditions such as smoking (Robinson et al., 2022), metabolic syndrome (Roy Chengappa et al., 2001; Bray et al., 2003) and a variety of psychiatric disorders including binge-eating and PTSD (post-traumatic stress disorder) (McElroy et al., 2003; Alderman et al., 2009). Furthermore, topiramate reduces neuronal dopamine activity in the mesolimbic cortical area and may therefore be useful in the treatment of alcohol use disorder due to a reduction of the rewarding effects of consumption (Weiss and Porrino, 2002; Johnson et al., 2004). Early randomized, double-blind, placebo-controlled clinical studies of topiramate in alcohol use disorder showed very promising results, with a significant reduction of alcohol consumption parameters and alcohol craving compared to placebo if topiramate was used as an adjunct to standardized anti-craving medication (Johnson et al., 2003a) or even as a first-line medication (Johnson et al., 2007). However, a subsequent randomized, placebo-controlled clinical study was not able to reproduce these results and did not find any significant effect of topiramate in the treatment of alcohol use disorder (Likhitsathian et al., 2013). To complicate matters further, a study of veterans with PTSD and comorbid alcohol use disorder reported that topiramate effectively reduced hyperarousal PTSD symptoms, as well as alcohol craving and consumption (Batki et al., 2014). In a meta-analysis including seven randomized placebo-controlled clinical trials of topiramate as a treatment for alcohol use disorder, a small to moderate overall effect in favor of topiramate was found, together with good results for abstinence and reduced heavy drinking, although not reaching significance for a reduction of alcohol craving (Blodgett et al., 2014). These promising results were confirmed in a Cochrane review, which showed a reduction of drinking parameters such as heavy drinking days and drinks per drinking day, as well as superiority in maintaining abstinence compared to placebo, even though craving reduction again did not reach significance. In addition to these hopeful clinical results, no difference in drop-out rate was found between topiramate and placebo despite the higher level of adverse events in the topiramate group (Pani et al., 2014). Topiramate has a relatively favorable adverse event profile, with mild to moderate reported symptoms mainly consisting of transient paraesthesia, anorexia, taste perversion, and memory impairment and concentration disorder (Johnson, 2005, 2008; Johnson and Ait-Daoud, 2010; Batki et al., 2014).

Conflicting or ambiguous study results, such as a failure to reach significance in the case of craving reduction, may be due to insufficient patient-therapy allocation. A pharmacogenetic study found that a single nucleotide polymorphism (rs2832407) in GRIK1, a gene encoding a subunit of the glutamatergic kainate GluK1 receptor, influenced the therapeutic outcome of topiramate. A patient subgroup carrying homozygous C alleles of rs2832407 showed a significantly greater response to topiramate as regards fewer heavy drinking days compared to other genotype subgroups (Kranzler et al., 2014a; 2014b, 2016). In another study the moderating effect of the rs2832407 genotype could unfortunately not be replicated in a prospective trial (Kranzler et al., 2021). On the other hand, further studies have shown the pharmacogenetic influence of single nucleotide polymorphisms (SNP’s), not only on treatment response but also on the post-treatment period following discontinuation of the medication (Kranzler et al., 2022). Therefore, introduction of personalized medicine, with consequent improved patient-therapy allocation, may deliver useful improvements in therapeutic responses. Furthermore, topiramate might also support concept anti-craving therapies as an adjunct medication, as shown in preclinical studies for combined treatment with ondansetron and topiramate (Moore et al., 2014).

Overall, the results to date for topiramate in the treatment of alcohol use disorder are very promising. Nevertheless, further clinical studies are needed in order to allow topiramate to be incorporated into existing therapeutic standards as a second-line or adjunct medication, a role in which it has shown great promise.

### 3.3 Ondansetron

Ondansetron is a selective serotonin 5-HT3 receptor antagonist with affinity for central as well as peripheral serotonin receptors in the gastrointestinal tract where it shows strong antiemetic effect. Ondansetron is now used to treat severe nausea and vomiting, especially after oncologic radiation therapy and chemotherapy, or in cases with opioid-induced postoperative nausea and vomiting (PONV). In contrast to other antiemetic medications, ondansetron shows no antidopaminergic or anticholinergic proprieties (Christofaki and Papaioannou, 2014).

Besides an antiemetic effect, ondansetron is an effective treatment for alcohol use disorder in patients with early onset alcoholism (EOA) (Soyka and Müller, 2017). A preliminary, double-blind, randomized, placebo-controlled study including healthy male volunteers addressed the psychological effects of alcohol ingestion. Pre-treatment with 4 mg oral ondansetron led to a significant reduction in the subjective pleasurable effect of alcohol, together with an attenuated desire to drink alcohol (Johnson et al., 1993). These initial findings were later confirmed in a large multicenter, double-blind, randomized placebo-controlled clinical trial that included 271 patients with diagnosed alcohol use disorder. Patients were assigned 1 µg/kg, 4 µg/kg or 16 µg/kg ondansetron twice a day or an identical placebo for a treatment duration of 11 weeks, with concomitant cognitive behavioral psychotherapy. Self-reported alcohol consumption was verified by quantitative measurement of carbohydrate-deficient transferrin (CDT) in plasma, a sensitive marker for alcohol consumption. Results showed a significant reduction of alcohol consumption in patients with EOA and superiority to placebo in terms of abstinence rate. A dosage of 4 µg/kg twice a day is particularly recommended for EOA (Johnson et al., 2000b). Interestingly, the therapeutic effect of ondansetron is only apparent in patients with EOA and fails to reduce craving or alcohol consumption in patients with late-onset alcoholism (LOA). This difference in therapeutic outcome is based on serotoninergic disturbance in EOA, which is regulated by ondansetron (Johnson et al., 2002; Kranzler et al., 2003).

Even without stratification for EOA or LOA, a placebo-controlled, double-blind clinical study of ondansetron 16 mg/d showed a modest but still significant reduction in heavy drinking days (Corrêa Filho and Baltieri, 2013). Besides an anti-craving effect, ondansetron has been shown to alleviate symptoms of anxiety, depression and hostility in patients with EOA (Johnson et al., 2003b).

In an effort to improve therapeutic outcomes, several clinical studies were conducted using pharmacological combination therapies such as ondansetron and naltrexone, and a significant superiority to placebo in reduction of craving, decreased automaticity of drinking and alcohol consumption was reported (Johnson et al., 2000a; Ait-Daoud et al., 2001a, 2001b).

Preliminary studies in rodents, with a focus on a combination of ondansetron and topiramate, have also shown promising results, especially as regards heavy drinkers (Lynch et al., 2011; Moore et al., 2014). In a within-subject, double-blind, placebo-controlled human laboratory study comparing ondansetron and sertraline, a genetic polymorphism of the serotonin 5-HT3 transporter (5-HTTLRP) was shown to be responsible for alterations in the effective strength of anti-craving ondansetron therapy. The results of this study demonstrated a direct interaction between a genetic polymorphism and a response to pharmacological therapy (Kenna et al., 2014a).

Several other studies have attempted to find genetic polymorphisms with predictive value for pharmacological treatment outcomes (Johnson et al., 2011, 2013; Kenna et al., 2014b; Müller et al., 2014; Hou et al., 2015; Thompson and Kenna, 2016). In a pharmacogenetic trial of ondansetron that included 251 participants with full genotype information, 118 specific genetic or other prognostic factors for therapy response were identified (Hou et al., 2015). A pharmacogenetic study looking at the association of genotypes with treatment responses found evidence that a five-marker genotype panel of single-nucleotide polymorphisms could predict effectiveness of ondansetron therapy (Johnson et al., 2013). Future progress in pharmacogenetics will allow better patient allocation to optimal pharmacological treatments.

In summary, ondansetron has shown promising results and could potentially be incorporated into standard therapies for treating alcohol use disorder, especially as a combination therapy with established anti-craving medicines (Soyka et al., 2017).

### 3.4 Varenicline

Varenicline is a derivate of the quinolizidine-alkaloid cytosine and acts as a partial agonist at the α4β2 and as a full agonist of α7 nicotinic acetylcholine receptors (nAChR) in the ventral tegmental area of the mesencephalon (Crunelle et al., 2010; Nocente et al., 2013). Varenicline is approved for smoking cessation in patients with nicotine dependence and is effective in the treatment of AUD and other substance dependences such as cocaine (McKee et al., 2009; Fucito et al., 2011; Hays et al., 2011; Childs et al., 2012; Mitchell et al., 2012; Plebani et al., 2012, 2013; Litten et al., 2013; Meszaros et al., 2013; Erwin and Slaton, 2014). In patients with alcohol use disorder, varenicline modifies endogenous dopamine pathways that are affected by exogenous substances and thus reduces alcohol-induced dopamine release in the nucleus accumbens. Although the precise mechanism of action is still unclear, inhibition of triggered dopamine release reduces rewarding effects and thereby disrupts maintenance of alcohol consumption (Ait-Daoud et al., 2006a, 2006b; Crunelle et al., 2010, 2011; Nocente et al., 2013). The 80%–85% comorbidity rate for nicotine dependence in patients with AUD illustrates the great potential of a medication able to treat both dependence diseases (Ait-Daoud et al., 2006a; Mitchell et al., 2012; Nocente et al., 2013).

For treatment of nicotine or alcohol use disorder, 0.5–1.0 mg of varenicline is administered twice daily and shows a nutrition-independent bioavailability of nearly 100%, facilitating rapid attainment of maximal plasma peak concentration. Varenicline shows only a negligible hepatic metabolism and few drug interactions (Foulds et al., 2006; Steinberg et al., 2011). The most often manifested drug side effects were gastrointestinal symptoms such as nausea, constipation or vomiting, but these were often only mild or intermediate in intensity. Further adverse effects, such as headache, insomnia, abnormal dreams and dry mouth were more often reported compared to placebo (Erwin and Slaton, 2014; Raich et al., 2016). Overall, varenicline can be considered safe, even in patients with psychiatric comorbidities, where no evidence was found for remarkable exacerbations or increased occurrence of adverse effects (Raich et al., 2016). In early studies, the anti-craving effect of varenicline was seen as a positive side-effect in patients treated for nicotine dependence, and even though the study population was a mix of alcohol-dependent and non-alcohol-dependent patients, the study results nevertheless showed a significant reduction of alcohol consumption in smokers treated with varenicline (McKee et al., 2009; Childs et al., 2012; Mitchell et al., 2012).

This observation could be reproduced in several preclinical studies on rats, which showed reduced alcohol consumption after administration of varenicline. Interestingly, pharmacological treatment seems to decrease the rewarding effect of alcohol rather than seeking behavior in rats, corresponding to similar cravings in humans, and lasted only as long as the substance was administered (Crunelle et al., 2010; Froehlich et al., 2017b; Czachowski et al., 2018).

Human clinical trials with 1 mg varenicline twice a day in alcohol-dependent patients gave similar results, producing a significant reduction of alcohol consumption (Litten et al., 2013; Meszaros et al., 2013; Plebani et al., 2013; de Bejczy et al., 2015). In one randomized, double-blind, placebo-controlled clinical trial, varenicline was found to be more effective in reducing craving and mood instabilities than in decreasing alcohol intake. Varenicline seemed to have a greater impact in patients with nicotine dependence comorbidity than in non-smokers (Plebani et al., 2013). In contrast, a large double-blind, randomized, placebo-controlled clinical study of 200 alcohol dependent participants showed a significant reduction in consumption of alcohol (heavy drinking days, drinks per day and drinks per drinking day) as well as reduced craving for alcohol. No difference was found between smokers or non-smokers (Litten et al., 2013).

Other double-blind, placebo-controlled clinical trials that included participants with the dual behavioral health risks of nicotine and alcohol-dependency showed that varenicline is an effective co-therapy for both comorbidities, reducing both drinking and smoking. Varenicline is an interesting new therapeutic option in that a single pharmacological substance impacts combined behavioral health risks (McKee et al., 2009; O’Malley et al., 2017; Hurt et al., 2018). Encouragingly, no direct correlation between the effects of varenicline on alcohol and nicotine consumption was found, indicating that varenicline can still be effective even when effects of the other comorbidity are absent (McKee et al., 2009).

Most studies of varenicline in AUD showed a significant reduction of alcohol craving (Roberts et al., 2017) and consumption, measured in heavy drinking days, drinks per day and drinks per drinking day, but did not show an increased abstinence rate. This could be due to the specific mechanism of interaction with the endogenous dopaminergic system, where varenicline decreases stimuli-triggered dopamine release and thus averts a rewarding alcohol effect (McKee et al., 2009; Fucito et al., 2011; Hays et al., 2011; Mitchell et al., 2012; Litten et al., 2013; Meszaros et al., 2013). On the other hand, not all studies were able to reproduce these results, and in some cases varenicline could not be shown to attenuate cue-induced alcohol craving relative to placebo (Miranda et al., 2020).

New therapeutic strategies tend to combine the advantages of varenicline with the established standard therapy for alcohol use disorder. In a study of rats carrying a genetic mutation conveying risk of alcohol use disorder, a combination of naltrexone and varenicline showed promising results that deserve further study (Froehlich et al., 2017a).

A preclinical double-blind, randomized, placebo-controlled study, including subjects who were both heavy drinkers and smokers, used functional neuroimaging (fMRI) to show the influence of combinations of pharmacological substances on neurological activity in region of interest (ROI) analysis and exploratory whole-brain analysis. The alcohol and nicotine dependent patients were either assigned to varenicline (2 × 1 mg) alone, naltrexone (25 mg) alone, a combination of naltrexone and varenicline or placebo. The study results showed a better response to combination therapy, suggesting that this could be a promising new strategy in AUD or comorbid alcohol-nicotine dependency (Ray et al., 2015).

In conclusion, varenicline appears to be a good alternative strategy, especially for patients with less-severe alcohol use disorder. In these patients it can reduce alcohol consumption and improve psychosocial functioning (Donato et al., 2021), and it may also be useful in patients with dual behavioral health risks due to alcohol and nicotine dependence. Thanks to these promising results and rare adverse effects, varenicline has a real chance to become a new standard therapy, particularly in combination with established therapies such as naltrexone or nalmefene (Erwin and Slaton, 2014; Soyka and Lieb, 2015; Soyka et al., 2017).

### 3.5 Antipsychotics

#### 3.5.1 Aripiprazole

Aripiprazole is an antipsychotic drug and is classified as an atypical neuroleptic for treatment of schizophrenia and manic or mixed episodes in bipolar-I-disorder. In some countries it is also approved for therapy of major depression. Aripiprazole is a partial agonist of dopamine D2 and serotoninergic 5-HT1A receptors, and an antagonist of serotonin 5-HT2A receptors, besides its effects on several other neurotransmitter systems in the central nervous system. Due to the involvement of dopaminergic mechanisms in the control of motivation, motivational behavior and reward control, they play a crucial role in the reinforcement of substance abuse and are therefore an interesting therapeutic target in alcohol use disorder (Brunetti et al., 2012).

In a preclinical study of an animal model with alcohol-preferring AA (Alko, Alcohol) rats, repeated treatment with aripiprazole significantly reduced alcohol drinking, while having no effect on the drinking of saccharine solution (negative control). However, aripiprazole dosages had to be quite high in order to reduce alcohol drinking and were therefore accompanied by side effects such as reduced locomotor activity (Ingman et al., 2006).

Reduction of alcohol consumption following aripiprazole treatment was observed in another animal study, and showed an higher striatal dopamine D2 receptor occupancy in case of alcohol consumption under aripiprazole treatment (Nirogi et al., 2013). In addition to these findings, in a preclinical study of prenatally stressed rats, an animal model for schizophrenia, aripiprazole led to a significant reduction in anxiety levels and even achieved effective anxiolysis (Ratajczak et al., 2016).

In a meta-analysis of antipsychotics in the treatment of primary alcohol use disorder, besides the mainly disappointing results for other antipsychotics, aripiprazole was associated with a significant reduction of alcohol consumption regarding heavy drinking days compared to placebo (Kishi et al., 2013). In a randomized, double-blind, comparison study of aripiprazole (5–15 mg) against the established anti-craving agent naltrexone (50 mg) over a study period of 16 weeks, craving reduction was found to be better with naltrexone treatment but alcohol abstinence was of longer duration with aripiprazole. By the end of the study there was no significant difference in relapse rate between the two groups and therefore aripiprazole and naltrexone were considered to be equally effective (Martinotti et al., 2009). In a review of aripiprazole in the treatment of alcohol use disorder, it was not only found to be effective for promoting alcohol abstinence, but also performed well in the reduction of craving, improved control of impulsive behavior and reduced alcohol-related psychic symptoms and anxiety (Martinotti et al., 2016).

On the other hand, a clinical study comparing aripiprazole against placebo reported negative findings. This multicenter randomized, double-blind, placebo-controlled study included 295 patients and had a treatment duration of 12 weeks. Despite aripiprazole titration from 2 mg/d up to a maximum dose of 30 mg/d at 28 days, no significant difference was found in abstinence rate between the aripiprazole and placebo groups. Furthermore, time to first drinking day and heavy drinking day rate were comparable between groups. Treatment-related side effects, as well as study discontinuation, were more common in the aripiprazole group. Even though these results were disappointing, alcohol consumption per drinking day decreased in the aripiprazole group, as did the subjective severity of dependence, and this was accompanied by greater subjective positive treatment effects with aripiprazole (Anton et al., 2008). Data on aripiprazole therefore remain insufficient and conflicting (Brunetti et al., 2012).

However, it seems likely that these conflicting results are a result of inadequate or incorrect patient-therapy allocation. In a study of the influence of impulsivity and self-control in patients with alcohol use disorder using aripiprazole, aripiprazole therapy was found to be especially beneficial for a subgroup of patients with poor self-control and high impulsivity (Anton et al., 2017). Similar results were found in a small randomized, double-blind, placebo-controlled study of non-treatment seeking alcohol-dependent patients, where aripiprazole was most effective in reducing alcohol consumption in the most impulsive patients with least self-control (Voronin et al., 2008).

These findings are further supported by a pharmacogenetic study of dopaminergic genetic variations. This study was posited on the strong interaction between dopamine regulation and substance abuse, and therefore considered the effect of variants on treatment outcomes of aripiprazole therapy for alcohol use disorder. VNTR polymorphisms (variable number tandem repeat polymorphism) in DAT1/SLC6A3, a gene coding for a dopamine transporter protein (DAT), as well as functional polymorphisms in COMT (catechol-O-methyltransferase), DRD2 (dopamine D2 receptor) and DRD4 (dopamine D4 receptor) were analyzed in 94 non-treatment seeking patients with alcohol use disorder. Following randomization to aripiprazole 15 mg/d or placebo, an fMRI alcohol-cue reactivity test and alcohol consumption test were conducted. Aripiprazole was found to reduce alcohol consumption and alcohol-triggered brain area activation in patients with a DAT1 9-repeat allele or in patients carrying a high number of variant alleles for DAT1, COMT, DRD2 and DRD4. All of these genetic variants are responsible for higher dopamine release and therefore stronger reward-related brain area activation. To summarize, these data have unequivocally demonstrated the influence of dopaminergic genetic variants on the probability of therapy success with aripiprazole. Therefore, aripiprazole seems to be a promising therapeutic strategy for patients with a genetic predisposition for elevated synaptic dopamine tone (Schacht et al., 2018).

Even though data are still conflicting and more placebo-controlled clinical trials and pharmacogenetic patient-allocation studies are needed, aripiprazole seems to have potential as a second-line therapy for patients with impulsivity and low self-control or for patients with a genetic predisposition.

#### 3.5.2 Quetiapine

Quetiapine is an atypical antipsychotic drug that undergoes multiple receptor interactions and modulates several neurotransmitter pathways. Quetiapine is not only an antagonist of serotonin 5-HT1A and serotonin 5-HT2A receptors, but is also an antagonistic of dopamine D1 and D2 receptors, the histamine H1 receptor and adrenergic α1 and α2 receptors. Quetiapine is approved for treatment of schizophrenia, bipolar disorder and unipolar depression. Besides these approvals, quetiapine is often used off-label in various therapy trials such as treatment of insomnia, and has been discussed as a possible treatment for alcohol use disorder (Ray et al., 2010).

However, while some smaller or open-label studies reported a decrease in craving and alcohol consumption (Martinotti et al., 2008; Ray et al., 2011; Brunette et al., 2016), most placebo-controlled studies or meta-analyses found no evidence that quetiapine is effective in the treatment of alcohol use disorder (Kishi et al., 2013). In two different randomized, double-blind, placebo-controlled clinical trials including patients with bipolar-disorder and alcohol use disorder, additional quetiapine titrated up to 600 mg/d over a study duration of 12 weeks had no effect on alcohol consumption (Brown et al., 2008, 2014). Although quetiapine did not influence alcohol consumption, a significant reduction in depressive symptoms was found in one of the studies (Brown et al., 2008). In the other study neither alcohol consumption nor depressive symptoms were reduced by quetiapine therapy (Brown et al., 2014). Similar results were found in another randomized, double-blind, placebo-controlled clinical trial of quetiapine fumarate XR in very heavy drinking alcohol-dependent patients. While quetiapine had no effect on craving or drinking, depressive symptoms and sleep disturbances were significantly reduced (Litten et al., 2012).

Due to the widespread off-label use or misuse of quetiapine for sleep disturbances, a study was initiated on the subject of sleep quality in alcohol-dependent patients undergoing quetiapine fumarate XR therapy and found improved sleep continuity and reduced insomnia (Chakravorty et al., 2014). In a secondary analysis of data from a clinical study on patients with alcohol use disorder, quetiapine was found to significantly reduce craving but only in a subgroup with comorbid insomnia (He et al., 2019).

A very interesting subgroup was identified in a randomized, double-blind, placebo-controlled clinical trial of quetiapine 400 mg/d in the treatment of Type A and Type B alcoholism. Type B alcoholism is defined as early-onset high severity dependence, with elevated psychopathology and treatment-resistance. While quetiapine had no influence on Type A alcoholism, a significant reduction of craving and alcohol consumption was found in a subgroup of patients with Type B alcoholism (Kampman et al., 2007).

Conversely, a double-blind, placebo-controlled study comparing combined quetiapine-naltrexone therapy with naltrexone alone did not find any additional effect of quetiapine on craving or alcohol consumption (Guardia et al., 2011). Furthermore, due to the widespread off-label use of quetiapine for sleep disturbances there are now serious safety concerns regarding the use and frequent misuse of quetiapine (Kim et al., 2017; Montebello and Brett, 2017).

To summarize, quetiapine shows little promise as a treatment for alcohol use disorder, even though it may be beneficial in certain subgroups. Most placebo-controlled clinical trials failed to produce any evidence supporting a beneficial role of quetiapine in craving reduction, reduction of alcohol consumption or in maintaining abstinence (Brown et al., 2008, 2014; Guardia et al., 2011; Litten et al., 2012). We can therefore firmly conclude that quetiapine has no application in the therapy of alcohol use disorder.

#### 3.5.3 Clozapine

Clozapine is an atypical neuroleptic with antipsychotic effects and multiple neurotransmitter circuit interactions where clozapine influences not only serotoninergic and dopaminergic neurotransmission, but also cholinergic, adrenergic and histaminergic transmission. Due to its superiority above other antipsychotic medications, clozapine is mainly used as second-line antipsychotic drug for treatment of therapy-resistant schizophrenia (Nucifora et al., 2017).

There is weak evidence derived from small retrospective studies that clozapine may have some benefit in the treatment of schizophrenia with comorbid substance use disorder, resulting in a reduction of alcohol consumption due to inhibition of dysfunctional brain reward circuits (Drake et al., 2000; Green, 2006; Kim et al., 2008). In a retrospective study of alcohol consumption in schizophrenia, patients treated with clozapine were more likely to remain abstinent than a group treated with risperidone (Green et al., 2003). In another non-randomized clinical study on patients with schizophrenia and substance use disorder, clozapine was more effective than other antipsychotic drugs in significantly reducing relapse risk (Brunette et al., 2006).

To date, clozapine has not been evaluated in a randomized, double-blind, placebo-controlled clinical study for treatment of alcohol use disorder, but only as a secondary treatment target in patients with schizophrenia. Although clozapine has beneficial effects when used for the treatment of schizophrenia with comorbid alcohol use disorder, the medication is certainly not a legitimate therapy for the treatment of primary alcohol use disorder.

#### 3.5.4 Other antipsychotic drugs

Several other antipsychotic drugs such as misulpride, flupenthixol decanoate, olanzapine and tiapride have been tested for use in the treatment of alcohol use disorder but have shown largely disappointing results (Kishi et al., 2013; Grunze et al., 2021). For better evaluation more studies should be conducted (Salloum and Brown, 2017).

### 3.6 Antidepressants

In patients with alcohol use disorder and comorbid depression, and vice versa, both psychiatric disorders require psychotherapeutic and pharmacological intervention because therapeutic progress or relapse in one may influence treatment outcomes of the other psychiatric disorder. Besides this obvious recommendation regarding treatment of the comorbidity and a very vague indication for SSRIs that may result in a small reduction of alcohol consumption, the overall evidence for use of antidepressants in the treatment of alcohol use disorder in non-depressed patients is very limited (Le Fauve et al., 2004; Nunes and Levin, 2004).

In a retrospective population-based cohort study, the use of selective serotonin reuptake inhibitors (SSRI’s) was associated with a reduction of hepatocellular carcinoma (HCC) risk in patients with alcohol use disorder in a cumulative dose effect manner (Chen et al., 2021). In another cohort study of patients with post-traumatic stress disorder (PTSD) and alcohol use disorder (AUD), the use of SSRIs resulted in a significant reduction of alcohol-related emergency department visits and alcohol-related medical hospitalization (Naglich et al., 2019). In a comparative study of noradrenergic versus serotonergic antidepressants on drinking and depressive outcomes for patients with alcohol use disorder (AUD) and co-occurring depression and/or PTSD, the study results showed that drinking outcome depended on the comorbid psychiatric disorder (Na et al., 2021). Beneficial effects of antidepressants for treatment of alcohol use disorder with co-occurring depression have been shown in several meta-analyses (Agabio et al., 2018). The antidepressants considered in the studies of this meta-analyses were amitriptyline, citalopram, desimipramin, doxepin, escitalopram, fluoxetine, fluvoxamine, imipramine, mianserine, mirtazapine, nefazodone, paroxetine, sertraline, tianepine, venlafaxine and vilofaxine (Agabio et al., 2018). In a meta-analysis of SSRIs for treatment of depression, anxiety and PTSD in patients with substance use disorder, a significant reduction was found for anxiety and depressive symptoms, in addition to a reduced alcohol craving and consumption (Fluyau et al., 2022). In contrast to the results of a meta-analysis of antidepressants in the treatment of substance use disorders, isolated treatment of alcohol use disorder in the absence of comorbid depression was not felt to be justified (Torrens et al., 2005).

These conflicting results could be due to the different treatment responses to serotonergic pharmacotherapy of type A and type B alcohol use disorder patients. In a placebo-controlled study with sertraline (200 mg/d over a 3-month treatment period), a significant effect was only detectable in type A alcohol use disorder, but not in type B (Dundon et al., 2004). Therefore, further preclinical and clinical studies, especially regarding serotonergic pharmacotherapy (e.g., SSRI) and differentiation by patient subtype such as type A versus type B alcohol use disorder, should be performed in the future (Pettinati et al., 2000; Kranzler et al., 2012b). Furthermore, pharmacogenetic differences may also play a role and deserve further consideration in studies of improved treatment allocation via personalized medicine (Kranzler et al., 2012a). Results for use of antidepressants in treatment of alcohol use disorder are still conflicting and needs further investigation, especially for better treatment allocation with differentiation for type A and B alcohol use disorder or depending on psychiatric comorbidity.

### 3.7 Lithium

Even though lithium, a monovalent cation from the group of alkali metals, is an effective treatment for bipolar disorder, depression (generally in combination with antidepressants), psychosis and schizophrenia, several studies and a meta-analysis did not find a significant effect of lithium on alcohol consumption and craving. Lithium is therefore not an effective treatment for alcohol use disorder (Lejoyeux and Adès, 1993), and no further investigations regarding alcohol use disorder have been undertaken.

### 3.8 Neuropeptides

Neuropeptides are endocrine neurosecretory peptide hormones and paracrine-acting co-transmitters in the central and peripheral nervous systems. Due to their neuromodulation of stress- and anxiety-related behavior and interactions with pathogenetic factors of alcohol use disorder, they may well be useful in the treatment of alcohol use disorder. Several neuropeptides have been identified as potential treatments of alcohol use disorder, including neuropeptide Y, corticotropin-releasing factor, neuropeptide S, and atrial natriuretic peptide (ANP) (Thorsell, 2010; Rodriguez and Coveñas, 2017; Hauser et al., 2020).

#### 3.8.1 Neuropeptide Y

Neuropeptide Y is involved in the modulation of several different effector systems, such as water and food intake regulation, control of mood and anxiety-related behavior, as well as vascular vasoconstriction and central autonomic functions. Neuropeptide Y acts on Y1-Y6 receptors, a group of at least six different G-protein coupled receptors, and co-secretion of neuropeptide Y with common neurotransmitters such as GABA, adrenaline and noradrenaline has a variety of modulating effects on neurotransmission (Grundemar and Håkanson, 1994; Boric et al., 1995). By activating Y1 and Y2 receptors in the central and basolateral amygdala, neuropeptide Y reduces the sensation of fear (Tasan et al., 2016). Due to the effects of neuropeptide Y on stress-level regulation, emotionality and mood control, as well as its anxiolytic properties as shown in several preclinical studies, together with its involvement in neurobiological responses to alcohol consumption and the pathogenesis of alcohol use disorder and withdrawal, neuropeptide Y may be a new treatment strategy not only for alcohol use disorders but also for depression and emotional and anxiety disorders (Heilig and Thorsell, 2002; Carvajal et al., 2006; Tasan et al., 2016).

In preliminary rodent studies, neuropeptide Y suppressed alcohol-induced inhibitory GABA release in central amygdala neurons and thus reduced the alcohol-reinforcement response in alcohol use disorder (Gilpin et al., 2011). The importance of neuropeptide Y for the pathology of this disease became evident in mice with a neuropeptide Y (NPY) gene deletion, as these mice exhibited high anxiety levels and a high alcohol-drinking phenotype (Gilpin, 2012). Furthermore, chronic alcohol consumption, as well as withdrawal, produces changes in neuropeptide Y release and Y receptor expression in the central nervous system such as the basal neuropeptide Y secretion deficits seen in the central amygdala of alcohol-preferring rats (Gilpin, 2012; Robinson and Thiele, 2017). In several preliminary rodent studies, infusion of neuropeptide Y, a Y1 receptor (Y1R) agonist and a Y2 receptor (Y2R) antagonist, into the central and extended amygdala significantly reduced alcohol consumption and binge-like ethanol drinking in treated rodents. Neuropeptide Y and its derivates therefore seem to be a promising new therapeutic strategy for reducing binge-like alcohol consumption, preventing progression from harmful alcohol use towards alcohol use disorder, as well as for the treatment of alcohol use disorder (Gilpin, 2012; Sparrow et al., 2012; Robinson and Thiele, 2017). In view of these promising results, more preclinical studies are needed in order to further evaluate the therapeutic potential of neuropeptide Y in the treatment of alcohol use disorder. Because no clinical study results are currently available, clinical studies in humans may also be justified.

#### 3.8.2 Neuropeptide S

Neuropeptide S is produced by neurons in many different human brain areas, but mainly in the amygdala region. Neuropeptide S binds specifically to the neuropeptide S receptor (NPSR), a G-protein coupled receptor (Reinscheid and Xu, 2005). Besides its stimulatory effect, expressed as reinforced wakefulness, hyperactivity and suppression of appetite, neuropeptide S also showed an anxiolytic effect in several rodent studies (Rizzi et al., 2008). In addition, neuropeptide S seems to play a role in the pathogenesis of alcohol use disorder and relapse risk after alcohol detoxification. In a rodent study, alterations in neuronal neuropeptide S receptor (NPSR) expression after alcohol intoxication were found, with an especially strong NPSR upregulation during alcohol withdrawal and prolonged abstinence. Furthermore, intracerebroventricular administration of neuropeptide S showed a more pronounced anxiolytic effect in alcohol-dependent rodents undergoing prolonged abstinence compared to control animals (Ruggeri et al., 2010). Genetic variants of NPSR1 in humans, such as the functional polymorphism p (Asn107lle) (rs324981, A>T), have a significant effect on alcohol consumption and risk of alcohol use disorder, effects that can be modified by sex, age and environmental factors (Laas et al., 2015). Taken together, these results suggest that the neuropeptide S regulation system and its receptors may be of interest in the treatment of alcohol use disorder. On the other hand, a study in which rodents were administered neuropeptide S found divergent results regarding alcohol seeking and consumption, with outcomes dependent on the genetic background of the rodent. Neuropeptide S may therefore only find use in the context of genetic variants predisposing to high alcohol consumption and, due to its anxiolytic activity, with comorbid high anxiety and depression-like symptoms (Cannella et al., 2016). Due to still limited data, no firm conclusions can be drawn at the moment, but further investigation of neuropeptide S and NPSR genetic variants in relation to alcohol use disorder appears justified.

#### 3.8.3 Corticotropin-releasing factor–antagonists

Corticotropin-releasing factor (or corticotropin-releasing hormone) is a peptide hormone involved in the regulation of the physiological stress response, and is released by neurons in the paraventricular nucleus of the hypothalamus. Corticotropin-releasing factor (CRF) binds two distinct corticotropin-releasing factor receptors, CRF1R and CRF2R, which can also bind other peptide ligands including three urocortins, Ucn1, Ucn2 and Ucn3, belonging to the corticotropin-releasing factor family (Ryabinin and Giardino, 2017). Preclinical data has demonstrated the relevance of the CRF signaling system to alcohol consumption and the pathophysiology of dependence, especially in relation to the transition from alcohol binge-drinking towards alcohol use disorder. As this appears to be related to the neuronal plasticity of CRF regulation, these neuroadaptative changes are a promising treatment target in alcohol use disorder (Phillips et al., 2015; Ryabinin and Giardino, 2017). Furthermore, genetic polymorphism of the CRF system have been linked to a human phenotype characterized by a risk of drug use that interacts with a stress stimulus or a stressful personal history, a risk especially relevant to alcohol use disorder (Zorrilla et al., 2014). Various antagonistic ligands of CRF receptors have been identified that can now be evaluated in preclinical studies for possible applications in the treatment of alcohol use disorder (Zorrilla et al., 2013). Indeed, preclinical studies have shown that CRF antagonists can significantly reduce emotionality, excessive drinking behavior and stress-induced alcohol seeking in animals with alcohol dependence (Heilig and Koob, 2007). Furthermore, a significant reduction of excessive alcohol consumption, as well a reduced relapse risk in case of occurrence of a stressful event, could be shown. Even though investigations have mainly focused on CRF1R antagonists, some evidence suggests that CRF2R may also be useful in the treatment of alcohol use disorder (Lowery and Thiele, 2010). However, despite promising preclinical results in animal models, translation to humans was not successful, as shown by initial small clinical studies. One double-blind, placebo-controlled clinical study of verucerfont (GSK-561,679), a CRF1 receptor antagonist, 350 mg/day, found no significant reduction of alcohol craving related to stress-, alcohol- or neutral stimuli. Besides a higher discontinuation rate in patients treated with verucerfont, no other clinical efficacy in the treatment of alcohol use disorder was evident (Schwandt et al., 2016). A randomized, double-blind, placebo-controlled clinical inpatient study, including 54 patients with alcohol use disorder, examined the CRF1 antagonist pexacerfont (BMS-562,086) at 300 mg/day for 7 days, followed by 100 mg/day 23 days, but found no effect on alcohol craving, emotional response or anxiety. According to the authors of the study, this lack of efficacy could be due to the fast dissociation of the antagonist from the receptor, and led to a proposal to evaluate slow-offset CRF1 antagonists in clinical trials (Kwako et al., 2015). Despite the discrepancies between preclinical and clinical studies, which may be due to study design or the pharmacokinetics of the antagonist in question, the CRF regulation system seems to play a crucial role in alcohol use disorder. Further investigations and preclinical and clinical trials are now needed in order to better evaluate the therapeutic potential of the CRF system as a treatment target for alcohol use disorder (Pomrenze et al., 2017; Schreiber and Gilpin, 2018). The role of neurobiological sex differences in alcohol use disorder also deserves more attention (Flores-Bonilla and Richardson, 2020).

#### 3.8.4 Oxytocin

The neuropeptide hormone oxytocin is mainly produced in the paraventricular nucleus of the hypothalamus and released by its neuronal axons in the posterior pituitary. Oxytocin plays a crucial role in childbirth during the peripartal and postpartal period, promoting uterine contraction and breastfeeding. It also has roles in social and sexual behavior, emotional control, and regulation of the stress response via the hypothalamic-pituitary-adrenocortical axis (Froemke and Young, 2021; Marsh et al., 2021). Besides these known effects, oxytocin may be useful in alcohol use disorder, potentially reducing alcohol craving and relapse risk by lowering anxiety and stress levels, as well as by reducing social withdrawal and enhancing prosocial behavior. The mechanism of action is still largely unknown, but may be due to inhibition of the interaction of growth hormone-releasing factor with GABAergic interneurons in the amygdala (Lee and Weerts, 2016; Faehrmann et al., 2018). The involvement of oxytocin in the pathogenesis of alcohol use disorder has been shown in post-mortem analysis of human brain, where disruption of the oxytocin regulation system was found (Lee et al., 2017). Another study on rat and human brain showed an upregulation of oxytocin receptor in the frontal and striatal brain area and a reduction of oxytocin expression in the hypothalamus of individuals with alcohol use disorder (Hansson et al., 2018). Furthermore, besides typical alcohol embryopathy, alcohol consumption during pregnancy may disrupt the fetal oxytocin system and therefore confer a prenatal risk for development of alcohol use disorder in later life (Holman et al., 2018). In light of these data, oxytocin seems to present an interesting new therapeutic opportunity.

In a study using a binge-like alcohol-drinking mouse model, oxytocin produced a dose-dependent reduction in alcohol-consumption of up to 45%, and appeared to reduce alcohol seeking behavior without altering general fluid intake (King et al., 2017). In another study of mice with alcohol use disorder, a dose-dependent reduction of alcohol seeking and reduced relapse risk upon stress stimulus was shown after intraperitoneal injection of oxytocin (King and Becker, 2019). These results were reproduced in a rat model that showed a significant reduction of alcohol consumption following intraperitoneal or intranasal application of oxytocin (Tunstall et al., 2019). Conversely, a study on prairie voles found a detectable reduction of alcohol consumption lasting only 1 h after oxytocin administration (Stevenson et al., 2017).

In contrast to the very promising preliminary results of animal studies, translation to human studies of alcohol use disorder has so far been difficult and has produced inconclusive or conflicting results. For example, one randomized, double-blind, placebo-controlled study reported a significant reduction of withdrawal symptoms with intranasal oxytocin (24 IE) as well as a reduced need for lorazepam (Pedersen et al., 2013). However, no such effect was detectable in another randomized, double-blind, placebo-controlled study (Mitchell et al., 2016). In a laboratory study of healthy social drinkers, oxytocin caused a reduction of functional connectivity in the nucleus accumbens visualized by fMRI during the alcohol-cue reactivity test, and had an inhibitory effect on craving (Bach et al., 2019). Furthermore, in a placebo-controlled clinical trial including patients with post-traumatic stress disorder (PTSD) and comorbid alcohol use disorder, intranasal oxytocin 40 IE caused a reduction of cortisol hormone reactivity during a stress test, but did not show any effect on subjective alcohol craving (Flanagan et al., 2019). In a randomized, double-blind, placebo-controlled clinical study of oxytocin in alcohol use disorder, patients showed a better social perception but no general reduction of craving. Nevertheless, if only a subgroup of patients with alcohol use disorder and comorbid anxiety disorder were considered, a significant reduction of alcohol craving was achieved (Mitchell et al., 2016). During no study were significant side effects reported concerning oxytocin compared with placebo, and a review on the safety and side effects of general use concluded that oxytocin has an excellent safety profile (MacDonald et al., 2011).

In conclusion, the failure to demonstrate an effect of oxytocin in humans in relation to treatment of alcohol use disorder, despite the very promising results from rodent studies, may be due to problems with study designs or due to the extremely short half-life of oxytocin (5–30 min). A reduction of craving and alcohol consumption may only be detectable shortly after oxytocin administration, as suggested by one rodent study (Stevenson et al., 2017). Despite these failures, oxytocin may still have considerable potential in the treatment of alcohol use disorder if pharmacokinetic problems can be solved (Ryabinin and Zhang, 2022), or if oxytocin could find use as an “as needed” medication for rapid reduction of alcohol consumption.

### 3.9 PF-05190457: Ghrelin receptor inverse agonist

Ghrelin is an orexigenic gastrointestinal peptide hormone, produced in neuroendocrine gastric parietal cells, with a role in the regulation of appetite, food intake, energy homeostasis and blood sugar control (Leggio, 2010; Farokhnia et al., 2018). Ghrelin also modulates reward and stress-regulatory pathways implicated in the pathogenesis of substance use disorder, suggesting that it may be useful in the treatment of alcohol use disorder (Zallar et al., 2017). Furthermore, several preclinical tests have shown a connection between the ghrelin regulation system and ethanol. Alcohol administration affects ghrelin serum levels and, inversely, a high ghrelin level leads to intensified alcohol craving. This finding points to a possible new pharmacological target in the treatment of alcohol use disorder (Leggio, 2010; Zallar et al., 2017). An inverse ghrelin receptor agonist, such as PF-05190457, which binds to the growth hormone secretagogue receptor (GHSR), may be useful for therapeutic purposes. Preliminary human pharmacokinetic and pharmacodynamic studies of PF-05190457, the first oral ghrelin receptor inverse agonist, showed that the compound is well tolerated in daily oral dosing and shows fast intestinal absorption, with plasma peak concentrations at 0.5–3 h and a half-life of 0.5–3 h following oral administration. PF-05190457 mediates the dose-dependent blockade of ghrelin action and leads to a reduction of gastric emptying and postprandial glucose levels. Besides a discrete elevation of heart rate of 13.4/min and occasional somnolence, PF-05190457 was well tolerated and did not show any severe adverse events (Denney et al., 2017). Furthermore, alcohol did not influence the pharmacological proprieties and plasma concentration of PF-05190457, and conversely, PF-05190457 did not alter alcohol absorption, concentration or elimination (Lee et al., 2020b). A placebo-controlled human laboratory study of heavy drinkers showed that PF-05190457 has no other endocrine effects and does not disturb any other blood hormone level (Lee et al., 2020a). In preclinical rodent studies, ghrelin administration increased alcohol consumption, whereas genetic ghrelin receptor knockout reduced alcohol consumption, while pharmacological ghrelin receptor blockade using PF-05190457 caused a significant reduction of alcohol craving and consumption (Farokhnia et al., 2019). In a placebo-controlled, single-blind, within-subject human laboratory study of heavy alcohol-drinking individuals, oral administration of 100 mg PF-05190457 significantly reduced alcohol craving during the alcohol cue-reactivity test (Lee et al., 2020b). Despite the very limited preclinical and clinical data, the ghrelin system seems to be a quite interesting potential target for further studies, and use of an inverse ghrelin receptor antagonist has shown promising preliminary results in the treatment of alcohol use disorder. Further preclinical rodent studies, as well as clinical studies, are needed to fully evaluate therapeutic potential.

### 3.10 NMDA receptor modulators

The NMDA receptors (N-methyl-D-aspartate receptors), a special subgroup of glutamate receptors, play a crucial role in regulation of alcohol drinking behavior. Several medications that modulate NMDA receptors activity are have been investigated for therapeutic use in alcohol use disorder (Vengeliene et al., 2005).

#### 3.10.1 Memantine

Memantine is a selective, non-competitive NMDA receptor antagonist, with moderate binding affinity, that also shows interactions with several other neurotransmitter systems. Memantine is approved for symptomatic treatment of Alzheimer’s disease. Besides its use in dementia, memantine has been investigated in relation to several other psychiatric disorders such as major depression, schizophrenia, bipolar disorder or anxiety disorder, mainly with disappointing or inconclusive results (Sani et al., 2012).

A variety of preclinical rodent studies of memantine for treatment of alcohol use disorder have been performed. These showed that memantine successfully reduces alcohol consumption but not impulsivity in alcohol-preferring mice (Oberlin et al., 2010), disrupts conditioned behavior to drug-related stimuli, (Vengeliene et al., 2015), and can reduce anxiety-like behavior of rats during alcohol withdrawal (Yuanyuan et al., 2018). In a rodent study of alcohol-dependent and non-dependent rats, memantine achieved a long-lasting (30 h) reduction in alcohol consumption in alcohol-dependent rats, as well as short-lasting (6 h) complete alcohol cessation in non-dependent rats. Because memantine seems to show a potentiation of alcohol effect but does not affect motivational behavior, this medication is unlikely to be suitable for the prevention of alcohol relapse but may have a role in alcohol-replacement therapy (Alaux-Cantin et al., 2015). In humans, memantine seems to be well tolerated, even in combined consumption with alcohol, and appears to mimic an alcohol-like effect (Bisaga and Evans, 2004).

Unfortunately, data from human clinical trials are very inconsistent. In a study of healthy, non-alcohol-dependent volunteers receiving memantine under electroencephalography (EEG) control, altered NMDA receptor function was found in non-alcohol-dependent volunteers with a positive family history of alcoholism versus volunteers without alcoholism in the family (Narayanan et al., 2013). Conversely, another placebo-controlled clinical study, using 20 mg/d memantine, found no effect of family history on treatment outcome. In this study, memantine was unable to reduce alcohol consumption despite a significant reduction in craving. Interestingly, patients with high impulsivity drank more alcohol under memantine treatment despite a subjective reduction in craving (Krishnan-Sarin et al., 2015).

Even in the case of craving, data is conflicting. While in the former study a significant reduction of craving was found (Krishnan-Sarin et al., 2015), in a randomized, double-blind, placebo-controlled clinical study comparing memantine 20 mg/d and 40 mg/d against placebo, a significant alcohol cue-induced craving reduction was evident, but no effect on craving was found prior to alcohol-cue stimulus under memantine treatment (Krupitsky et al., 2007).

Divergent results have also been reported concerning alcohol consumption and abstinence. While one open-label study (without a placebo control) of memantine as an supplementary treatment for bipolar disorder with comorbid alcohol use disorder found a significant reduction in alcohol consumption (Lee et al., 2018), other studies have reported an inverse effect. In a randomized, double-blind, placebo-controlled treatment trial of memantine 40 mg/d versus placebo in 34 patients with alcohol use disorder (duration 16 weeks), memantine showed no effect at all. Conversely, a significantly higher abstinence rate and fewer heavy drinking days were noted in the placebo group. Apart from significantly more side effects, memantine failed to show any other effect (Evans et al., 2007).

In conclusion, memantine appears to have no application in the reduction of alcohol consumption or in the general treatment of alcohol use disorder. However, memantine might find a use in psychotherapeutic alcohol-cue stimuli deconditioning therapy that aims to pharmacologically disrupt conditioned behavior due to drug-related stimuli. Although further clinical trials are justified, memantine is clearly not useful as a classic anti-craving drug.

#### 3.10.2 Ifenprodil

Ifenprodil is a non-competitive NMDA receptor antagonist with particular binding affinity for the GluN1 and GluN2B subunits (glycine-binding NMDA receptor subunit), as well as a G-protein-activated inwardly rectifying potassium (GIRK) channel inhibitor (GIRK inhibitor). Furthermore, ifenprodil interacts with several other neurotransmitter regulation systems and receptors such as alpha1-adrenergic receptors, serotonin receptors and sigma receptors (Kobayashi et al., 2006).

In an early rodent model study, ifenprodil successfully suppressed the severity of alcohol withdrawal and reduced alcohol-related behavioral changes in mice, which led to proposed clinical trials for treatment of alcohol use disorder (Malinowska et al., 1999).

Similar results were found in a study on NMDA receptor antagonists, where ifenprodil has shown to effectively reduce relapse-related behavior in long-term alcohol drinking rats during an alcohol deprivation experiment (Vengeliene et al., 2005). In a prospective, randomized, controlled, rater-blinded study of 68 patients with alcohol use disorder, ifenprodil 60 mg/d for 3 months significantly reduced both alcohol consumption and heavy drinking days (Sugaya et al., 2018).

In order to evaluate the clinical applicability and effectiveness of ifenprodil in alcohol-dependent patients, further randomized, double-blind, placebo-controlled clinical trials are needed.

#### 3.10.3 Other NMDA receptor modulators

Several other NMDA receptor modulators such as hemantane (Kolik et al., 2017) or ketamine (Jones et al., 2018) have been preclinically or clinically tested in alcohol use disorder or are still under investigation. Hemantane, a low-affinity non-competitive NMDA-receptor antagonist, was evaluated in DBA/2 mice model of alcoholism and was found to attenuate alcohol-seeking behavior after acute withdrawal (Kolik et al., 2017). In a systematic review on ketamine in treatment of substance use disorder, data suggests ketamine to facilitate abstinence across multiple substances, as well as in alcohol use disorder (Jones et al., 2018). For better evaluation more studies should be conducted (Ivan Ezquerra-Romano et al., 2018).

### 3.11 Opioid system modulators

Besides the already well-established first-line anti-craving opioid receptor antagonists naltrexone and nalmefene, several other substances with opioid receptor activity or interactions with the endogenous opioid system are under investigation for treatment of alcohol use disorder (Hillemacher et al., 2011).

#### 3.11.1 Samidorphan

Samidorphan (ALKS-33) is a μ-opioid receptor antagonist developed and investigated for the treatment of various psychiatric disorders such as major depression, schizophrenia, binge-eating disorder and alcohol use disorder (Chaudhary et al., 2019).

While the medication shows promising effects for treatment of major depression, especially as a combination product (ALKS-5461) of buprenorphine/samidorphan, (Ehrich et al., 2015), the results with binge-eating disorder were disappointing, showing no significant effect of samidorphan (McElroy et al., 2013). Due to its favorable side effect profile, where somnolence was found to be the most common treatment-related adverse event, together with a small abuse risk, samidorphan is an interesting candidate for clinical trials on alcohol use disorder (Turncliff et al., 2015; Pathak et al., 2019). In a phase 2, randomized, double-blind, placebo-controlled clinical trial, patients with alcohol use disorder were treated with samidorphan (1, 2.5, 10 mg/d or placebo) over a study duration of 12 weeks. Even though the primary endpoint, the percentage of subjects with no heavy drinking days, did not show any difference compared to placebo, samidorphan treatment led to a significant reduction of alcohol craving and consumption, as well as a lower drinking risk level (O’Malley et al., 2018). For treatment of schizophrenia with comorbid alcohol use disorder, a phase 2, randomized, double-blind clinical trial of olanzapine-samidorphan combined therapy is still ongoing (Citrome et al., 2019).

In order to evaluate the therapeutic potential of samidorphan as an anti-craving drug in the treatment of alcohol use disorder, as well as to compare it to established therapeutic options, further randomized, placebo-controlled, clinical trials are needed.

#### 3.11.2 Ondelopran

Ondelopran (LY2196044) is a non-selective opioid receptor antagonist that interacts with endogenous opioid-mediated reward pathways and was therefore developed for treatment of alcohol use disorder.

In order to assess its anti-craving potential, a multicenter, randomized, double-blind, placebo-controlled phase 2 clinical trial was conducted over a period of 16 weeks with 375 treatment-seeking patients with alcohol use disorder. The patients were either treated with LY2196044 at dosages of 125 mg/g or 150 mg/d, or received placebo. Even thought there was no significant difference in the percentage of heavy drinking days between treatment or placebo groups, a significant reduction of alcohol consumption (number of drinks per day) was found.

An even better response to LY2196044 treatment was detected in patients who were previously identified as carriers of a genetic VNTR-L (variable number tandem repeat) in the dopamine D4 receptor, a receptor mainly located in frontal lobe, hippocampus and amygdala. In this study the safety profile of LY2196044 was evaluated and found to be equivalent to other opioid receptor antagonists (Wong et al., 2014).

In contrast, pharmacokinetic-pharmacodynamic investigations with physiologically-based absorption modeling found that LY2196044 shows a very high intersubject variability. The variability of plasma concentration time profiles in the absorption phase could not be explained by prior *in vitro* measurements. It was therefore concluded that the negatively-affected permeability and gastrointestinal transit was likely due to opioid receptor antagonism of intestinal receptors by the drug itself, explaining high intersubject variability (Ding et al., 2013).

To date, no further investigations or clinical trials have been undertaken to re-evaluate possible clinical use of LY2196044 for treatment of alcohol use disorder.

### 3.12 Vasopressin receptor antagonists

#### 3.12.1 ABT-436

ABT-436 is a potent and orally-active arginine vasopressin type 1B receptor antagonist (V1B antagonist) with high binding selectivity. ABT-436 was developed for the treatment of anxiety disorders, major depression and alcohol use disorder due to its modulatory effect on HPA axis activity and stress level regulation.

Evaluating its anti-depressive properties, a randomized, placebo-controlled, phase 1b clinical trial showed rather disappointing results, finding significantly more drug-related adverse effects than placebo but with no measurable anti-depressive effect according to the Hamilton Depression Rating Scale (HAM-D-17) (Katz et al., 2017). Despite its impact on blood pressure and pulse rate regulation, as well as causing several HPA axis hormone interactions, ABT-436 was found to be safe for use in humans (Katz et al., 2016a). ABT-436 also showed no pharmacokinetic or pharmacodynamic interactions with alcohol (Katz et al., 2016b).

In a phase 2, randomized, double-blind, placebo-controlled 12-week clinical trial including 150 patients with alcohol use disorder, ABT-436 was titrated up to 800 mg/d for evaluation of impact on alcohol consumption. While the primary study outcome, percentage of heavy drinking days, was lower, it did not reach statistical significance upon ABT-436 treatment compared to placebo, whereas the secondary endpoint, the percentage of abstinent days, significantly increased with ABT-436 treatment. Unfortunately, no other alcohol drinking measure, alcohol craving or alcohol-related outcome differed between the therapeutic and placebo group. In a subgroup analysis, the medication showed a better performance for high stress level alcohol-dependent patients (Ryan et al., 2017).

Probably due to these not entirely convincing results, further development and testing beyond phase 2 was discontinued.

#### 3.12.2 SSR149415

SSR149415 is a selective and orally-active non-peptide vasopressin type 1B receptor antagonist (V1B antagonist) that has shown anxiolytic and antidepressant effects in preliminary rodent model trials (Griebel et al., 2002; Overstreet and Griebel, 2005). In an animal model of alcohol-preferring rats, the stress-responsive arginine vasopressin (AVP)/V1B receptor system was found to play a crucial role in the regulation of alcohol-drinking behavior. SSR149415 blocked stress-induced drug-seeking behavior in treated rats and therefore caused a significant reduction of alcohol consumption (Zhou et al., 2011). In another rodent model of excessive alcohol-drinking mice, SSR149415 was again shown to reduce alcohol consumption and was furthermore tested in combination with the established anti-craving medication naltrexone. The combined administration of SSR149415 and naltrexone reduced alcohol consumption much more efficiently, and at therapeutic doses lower than the individual single doses needed to achieve the same reduction in consumption. SSR149415 has therefore proven effective for treatment of excessive alcohol drinking in a mouse model, and has also shown a synergistic effect in co-therapy with naltrexone (Zhou et al., 2018).

Nevertheless, clinical trials have never been conducted in humans and further investigation and development of medications has been discontinued.

### 3.13 Mifepristone

Glucocorticoid hormones such as cortisol play a crucial role not only in the dysregulation of stress-response regulating systems like the hypothalamic-pituitary-adrenal (HPA) axis, they also play a crucial role in the genesis of several psychiatric diseases (Howland, 2013). This led to the evaluation of the progesterone and type II glucocorticoid receptor antagonist, mifepristone, for potential in the therapy of alcohol use disorder (Vendruscolo et al., 2012; Repunte-Canonigo et al., 2015). In several preclinical studies of a rat model of alcohol use disorder, administration of mifepristone significantly reduced alcohol withdrawal symptoms and hyperexcitability (Jacquot et al., 2008; Sharrett-Field et al., 2013) as well as reducing alcohol-induced neurodegeneration, resulting in lower memory deficits (Jacquot et al., 2008; Cippitelli et al., 2014). Furthermore, a dose-dependent reduction of voluntary alcohol consumption following pre-treatment with mifepristone was shown in another rodent study, whereas pre-treatment with the mineralocorticoid receptor antagonist spironolactone had no effect (Koenig and Olive, 2004). In contrast to these promising results, a non-human primate study of the effects of mifepristone on alcohol-seeking and self-administration in adult baboons produced very disappointing results. Not only did the pharmacokinetics of mifepristone prove non-linear and appeared capacity-limited, no effect on alcohol seeking and consumption behavior was noted (Holtyn and Weerts, 2019). On the other hand, a double-blind, placebo-controlled clinical trial with 56 alcohol-dependent patients found that mifepristone 600 mg/d was effective in the reduction of alcohol cue-induced craving and alcohol consumption during the 1-week treatment and 1-week post-treatment phase (Vendruscolo et al., 2015). Despite the small number of participants and short study duration, the results are interesting and deserve further investigation. Indeed, new and larger studies of mifepristone are currently being conducted (NCT02243709 and NCT02989662) (Donoghue et al., 2016).

Even though some study results seem promising and deserve further investigation, concerns regarding misuse of mifepristone should be considered when determining clinical applicability. In gynaecology, the progesterone antagonist effect of mifepristone is exploited to achieve drug-mediated abortion of pregnancy, and at the same dosage (600 mg) used in the treatment of alcohol use disorder. Uncontrolled supply of mifepristone to the alcohol-dependent, with the concomitant risk of unintended use, would lead to a significant rise in illegal abortion of pregnancy. Therefore, this drug should only be taken under visual supervision of a medical expert.

### 3.14 Ibudilast

Ibudilast is a non-selective phosphodiesterase (PDE) inhibitor that mainly inhibits the PDE4 subtype, but also acts on PDE3, PDE10 and PDE11. Its anti-inflammatory property is based on suppression of pro-inflammatory cytokines, upregulation of anti-inflammatory cytokines and interaction with several other immune-active cells and transmitters. Ibudilast is mainly used for treatment of bronchial asthma (Schwenkgrub et al., 2017).

In several studies, drugs of abuse have been found to induce neuroinflammatory signals through interaction with microglia and astrocytes and therefore disrupt glutamate homeostasis (Bachtell et al., 2017). Due to its anti-neuroinflammatory properties, ibudilast has been proposed and investigated for treatment of substance use disorders (Crews et al., 2017; Schwenkgrub et al., 2017; Kohno et al., 2019).

The ability of ibudilast to reduce alcohol consumption has been investigated in several preliminary rodent studies, where it was shown that heavy-alcohol drinking rats reduce alcohol consumption by approximately 50% with dosages that did not affect non-dependent animals (Bell et al., 2015).

In humans, ibudilast was first tested in a randomized, double-blind, placebo-controlled clinical study of non-treatment seeking patients with mild to severe alcohol use disorder. Participants received 50 mg ibudilast twice a day or a corresponding placebo, but unfortunately no medication effect was found regarding subjective responses to alcohol stimulus such as a reduction of alcohol craving. In a secondary data analysis for depressive symptomatology, ibudilast was found to significantly reduce alcohol craving (Ray et al., 2017).

Even though the available evidence is still weak and only one randomized, double-blind, placebo-controlled study has been conducted in humans, further investigation of the use of ibudilast in the treatment of alcohol use disorder seems worthwhile, especially in patients with comorbid depressive symptomatology.

### 3.15 Citicoline

Citicoline (cytidine 5′-diphosphocholine) is an intermediate product in the biosynthetic pathway of structural cell membrane phospholipids such as phosphatidylcholine, a major component of biological membranes. Due to its importance in neuronal cells, citicoline increases brain metabolism and interacts with several different neurotransmitters, leading to an increase of dopamine levels in the central nervous system. Furthermore, citicoline influences neuronal membrane Na+/K + ATPase and mitochondrial ATPase, as well as inhibiting cellular apoptosis and potentiating neuroplasticity (Secades and Lorenzo, 2006).

In addition to many other therapeutic applications, citicoline has been investigated as a treatment for substance use disorder (Wignall and Brown, 2014; Shen, 2018b). In various randomized, double-blind, placebo-controlled clinical studies of patients with bipolar disorder and comorbid cocaine addiction, citicoline was shown to significantly reduce cocaine consumption, although in one study a habituation effect with continual effect reduction was shown (Brown et al., 2007, 2015). Citicoline has also been tested in other substance use disorders, such as methamphetamine dependence with comorbid bipolar or unipolar depression, although the drug did not lead to a reduction of methamphetamine consumption (Brown and Gabrielson, 2012).

As regards alcohol use disorder, citicoline was clinically investigated in a randomized, double-blind, placebo-controlled trial with 62 patients over a study duration of 12 weeks. After randomization, patients were treated with citicoline 2000 mg/d or placebo and underwent several tests for alcohol consumption and craving, as well as other neurological and psychiatric examinations. Regrettably, citicoline failed to show any effect on alcohol consumption, craving or depressive symptoms (Brown et al., 2019).

In summary, while clinical evidence is perhaps still insufficient, at present citicoline seems to be both inappropriate and ineffective for treatment of alcohol use disorder.

### 3.16 Endocannabinoid system modulators

The endocannabinoid system is a receptor-ligand signaling system triggered by endogenous lipid transmitters derived from arachidonic acid, such as arachidonylethanolamide (AEA), 2-arachidonylglycerol (2-AG), 2-arachidonylglycerol ether (noladin ether) and O-arachidonoyl ethanolamine (O-AEA, virodhamine).

Endocannabinoids are synthesized and released upon demand by receptor-activated stimuli and act on G-protein-coupled cannabinoid receptors on the target cell. Cannabinoid receptor 1 (CB1R) is expressed in the central nervous system, mainly on cerebellar and hippocampal neurons, as well as basal ganglia. CB1R is therefore not only important for motor control but is also strongly associated with emotional control mechanisms and motivational behavior. CB1R is also expressed in the peripheral nervous system in the gastrointestinal tract where it plays a role in autonomic regulation, and further in many different tissues such as pancreas, liver, adipose tissue, heart muscle, skeletal muscle and the reproductive system (Mouslech and Valla, 2009). In the central nervous system, CB1R is present on excitatory neurons as well as inhibitory interneurons and is therefore not only important to postsynaptic transmission but also has a crucial role in retrograde signaling in glutamatergic and GABAergic synapses. Furthermore, endocannabinoids interact with many different neurotransmitters and signaling pathways. Cannabinoid receptor 2 (CB2R) is present in several neuronal subpopulations, as well as in astrocytes, microglia cells and immune cells, where it helps regulate immune system function (Basavarajappa, 2019). Endocannabinoid reuptake into neuronal cells is performed by a specialised endocannabinoid uptake system, leading to inactivation and degradation by the enzymes monoacylglycerol lipase (MAGL) and fatty acid amide hydrolase (FAAH) (Basavarajappa and Hungund, 2005; Rodríguez de Fonseca et al., 2005; Mouslech and Valla, 2009; Basavarajappa, 2019).

Because the endocannabinoid system is important for regulation of emotional control and motivational behavior, modulation of these pathways may well provide an interesting starting point for development of new medications for alcohol use disorder (Basavarajappa and Hungund, 2005; Mouslech and Valla, 2009; Maccioni et al., 2010; Turna et al., 2019).

#### 3.16.1 Rimonabant

Rimonabant (SR141716) is a selective CB1 receptor blocker effective in the treatment of obesity, tobacco smoking and cardiometabolic risk factors such as hyperlipidaemia and hyperglycaemia in diabetes (Curioni and André, 2006; Gelfand and Cannon, 2006).

Because rimonabant can significantly reduce cigarette smoking and improve abstinence rate compared to placebo, similar effects might be expected with other substance use disorders (Gelfand and Cannon, 2006). In several preclinical studies, rimonabant reduced alcohol-related behaviors in mice and rats such as alcohol seeking, drinking and self-administration (Colombo et al., 2007).

Despite these promising preliminary results, rimonabant failed to show any significant effect on alcohol consumption in the only human clinical study with a therapy target of abstinence. In this 12-week, randomized, double-blind, placebo-controlled study of 260 patients with alcohol use disorder, 2 × 10 mg/d rimonabant showed only a minimal, non-statistically significant reductions in the relapse rate (41.5% vs. 47.7% with placebo) and relapse to heavy drinking (27.7% vs. 35.6% in placebo group). This study also reported good tolerability but slightly higher depression-related adverse events (3.8% rimonabant group vs. 1.6% in placebo group) (Soyka et al., 2008). In a small, randomized, double-blind, placebo-controlled study on non-treatment seeking heavy alcohol drinkers, rimonabant 20 mg/d failed to show any effect on alcohol consumption (George et al., 2010).

Due to suspected safety risks reported in several clinical studies, especially those concerning severe depression-related adverse events, anxiety disorders and suicidal tendencies, rimonabant had to be withdrawn from clinical use (Gelfand and Cannon, 2006; Steinberg and Cannon, 2007; Maccioni et al., 2010).

Even though rimonabant failed in the treatment of alcohol use disorder, the knowledge gained concerning depression-related side effects has suggested the endocannabinoid system as a target for the development of novel anxiolytic and antidepressant drugs (Gaetani et al., 2009).

#### 3.16.2 Surinabant

Surinabant (SR147778), a CB1 receptor antagonist similar to rimonabant, led to a significant reduction of alcohol consumption in preclinical studies of alcohol-dependent rats (Gessa et al., 2005; Lallemand and De Witte, 2006). Compared to rimonabant, surinabant led to an even more dramatic decrease in alcohol consumption in alcohol-dependent rats and showed a longer-lasting effect in a free drinking choice experiment (Lallemand and De Witte, 2006). Due to the withdrawal of rimonabant following recognition of anxiety symptoms and depression-related adverse events, clinical trials of surinabant in alcohol use disorder have never been conducted. However, a randomized controlled clinical study of surinabant in smoking cessation reported no improvement of smoking cessation rate, but did report elevated adverse side effects such as insomnia and anxiety symptoms in the surinabant group compared to the placebo group (Tonstad and Aubin, 2012).

Although no clinical trial data on surinabant in alcohol use disorder are available, we suspect that the anxiety and depression-related adverse event profile of surinabant would resemble that of rimonabant. Further clinical trials of surinabant for alcohol use disorder are therefore unlikely to yield promising results.

#### 3.16.3 AM4113

The new CB1 receptor neutral antagonist, AM4113, has proven to be effective for treatment of tobacco and cannabis dependence in a squirrel monkey model, without showing the severe side effects evident for rimonabant (Schindler et al., 2016).

In a preclinical study using nicotine-dependent rats, AM4113 not only showed an absence of anxiety and depression-related adverse events, but even seemed to have an antidepressant-like effect (Gueye et al., 2016). In another preclinical study of binge-drinking mice, pretreatment with AM4113 led to a reduction of alcohol-induced dopamine release in the nucleus accumbens, as well as a significant suppression of alcohol consumption in a free choice two-bottle experiment (Balla et al., 2018). Clinical studies of patients with alcohol use disorder have not been conducted to date. Due to the promising preclinical results, together with the absence of anxiety and depression-like side effects and perhaps even an antidepressant effect, further preclinical and clinical investigations for treatment of alcohol use disorder are recommended.

#### 3.16.4 Other endocannabinoid system modulators

Many other medications with specific binding affinities for or modulating effects on endocannabinoid receptors are being developed or are under investigation (Vasiljevik et al., 2013).

For example, the diacyl glycerol lipase inhibitor DO34 reduces alcohol searching behavior and habitual responses to ethanol, likely due to the inhibition of endocannabinoid 2-arachidonoyl glycerol (2-AG) biosynthesis. Similar results were found following administration of the cannabinoid receptor type 1 antagonist AM251 and the endocannabinoid transport inhibitor AM404 (Gianessi et al., 2019).

In another study, URB597, an inhibitor of fatty acid amide hydrolase (FAAH), causes an increase in the brain level of endocannabinoid anandamide (AEA) and therefore protects the brain of adolescent rats from alcohol-induced neuromodulation and neurotoxicity due to chronic alcohol consumption (Bellozi et al., 2019). Furthermore, URB597 does not show the full profile of behavioral responses, such as anxiety and depression-like symptoms, found with modulators that have a direct affinity for CB1 receptors, e.g., rimonabant. The elevated level of brain AEA caused by URB597 even shows an anxiolytic and antidepressant effect (Gaetani et al., 2009).

Two new aminoalkylindole derivates, TV-5-249 and TV-6-41, have proven effects on alcohol-dependent mice due to their dual action as CB1 receptor antagonists and CB2 receptor agonists, mediating a reduction of alcohol abuse-related behavioral responses in one study. This preclinical study also reported differences in adverse side effects regarding TV-5-249 and TV-6-41 compared to rimonabant (Tai et al., 2018).

Another promising future therapy concept is the use of dual receptor ligands for cannabinoid targets, such as NF 10–360, a dual PPARα/γ agonist, and OLHHA, a dual CB1 receptor antagonist/PPARα agonist. In a preclinical study in a rat model, both drugs achieved a significant reduction of alcohol intake and alcohol self-administration, and with an absence of toxicological effects or tolerance effects following repetitive administration of the drug (Alen et al., 2018).

If these new endocannabinoid system modulators are to be used in future therapies of alcohol use disorder, further investigation is needed, including preclinical trials and clinical studies on alcohol-dependent patients.

### 3.17 Gamma-hydroxybutyrate (GHB)

Gamma-hydroxybutyrate (GHB, also known as gamma-hydroxybutyric acid) is an endogenous neurotransmitter with a depressant action on the central neural system. It is also a psychotropic relaxant with a euphoric effect attributable to its strong affinity for GHB receptors on pre- and postsynaptic neurons, as well as partial agonism at GABA-B receptors (Schep et al., 2012; Brennan and Van Hout, 2014). Due to its binding of synaptic GABA-B receptors and extra synaptic GABA-A receptors, GHB shows an alcohol mimetic effect (van den Brink et al., 2018).

Whereas GHB is directly bioactive, its precursors, gamma-butyrolactone (GBL) and 1,4-butanediol (1,4-BD), first need bioenzymatic transformation into active GHB. After oral administration, GHB shows rapid intestinal absorption and reaches peak plasma concentrations within 1 h. Due to the relatively small distribution volume and a fast passage across the blood-brain barrier, GHB produces a relatively fast narcotic-psychotropic effect after administration. Fast hepatic metabolism and rapid elimination means that GHB has a short duration of action, with a half-life of 20–60 min (Schep et al., 2012).

Because of the narcotic and psychotropic effect, GHB and GBL (“liquid ecstasy”) are known drugs of abuse and have achieved notoriety due to an association with drug-facilitated sexual assault (“date-rape drug”) (Grela et al., 2018). Sodium oxybate is the sodium salt of gamma-hydroxybutyrate, and in contrast to its abusive use, has been shown to be effective in treatment of narcolepsy and cataplexy, as well as nocturnal sleep disturbances (Abad, 2019).

Sodium oxybate is approved as a liquid formulation (Alcover) in Italy and Austria for therapy of alcohol withdrawal syndrome (AWS) and in relapse prophylaxis of alcohol use disorder for the maintenance of abstinence (Keating, 2014). In a randomized, double-blind, comparative study of alcohol withdrawal syndrome, sodium oxybate was shown to be as effective as oxazepam, the gold standard benzodiazepine in AWS treatment. Furthermore, sodium oxybate has proven to be safe, with good tolerability and an absence of significant side effects (Caputo et al., 2014).

Sodium oxybate is also effective in relapse prophylaxis of patients with alcohol use disorder. Several studies have shown a significant reduction of craving, as well as an increase in abstinence rate (Keating, 2014; Skala et al., 2014; Mannucci et al., 2018). Another meta-analysis found that sodium oxybate was superior to disulfiram and placebo in craving reduction and had an even stronger effect on abstinence maintenance than naltrexone and disulfiram (Leone et al., 2010).

Conversely, sodium oxybate did not influence abstinence in heavy drinkers when given as a monotherapeutic medication (Mannucci et al., 2018).

Investigations of a combination of sodium oxybate with established relapse prophylaxis therapy protocols have been reported. Administration of additional nalmefene, 18 mg as needed, in sodium oxybate non-responder patients found that craving and alcohol consumption were reduced and abstinence improved. Nalmefene seems to have a modulating impact on the excessive reward effect of sodium oxybate and therefore reduces alcohol craving and persistent alcohol intake, as well as sodium oxybate craving (Caputo et al., 2016).

Despite these promising results, serious safety concerns due to the very high addiction and abuse risk of sodium oxybate induced the WHO to advise against the widespread use of GHB in the treatment of alcoholism (World Health Organization, 2012b; 2012a). Besides risks of illicit administration in order to commit crimes, GHB shows a high addiction potential and is often abused due to its psychotropic effects. GHB also shows dramatic potentiation of neuro-depressant effects in combination with alcohol ingestion, leading to a severe reduction in consciousness. This combination has led to considerable resistance to use of sodium oxybate without strict regulation and control (Miró et al., 2017; Galicia et al., 2019).

Despite these serious reservations, sodium oxybate has been under investigation in Italy and Austria for more than 25 years and has produced excellent results, especially in patients with a very high drinking risk in whom a major reduction in relapse was noted due to a strong reduction of craving and maintenance of abstinence. Adverse side effects were very rare, even after testing in more than 260,000 alcohol-dependent patients, and few cases of GHB abuse were reported. A summary of several meta-analyses therefore not only considered sodium oxybate to be effective in the treatment of alcohol withdrawal syndrome and maintenance of abstinence during relapse prevention treatment, but also well-tolerated and safe in applications related to alcohol use disorder (van den Brink et al., 2018).

With strictly-controlled administration to prevent abuse, sodium oxybate could potentially become an established second-line therapy in patients who show an insufficient response to current anti-craving therapies. Further investigations of safety and reduction of abuse risk are needed however.

## 4 Summary, interpretation and outlook

As shown in detail above, several different drugs and medication classes have been investigated for their potential use in the treatment of alcohol use disorder, a clinical area where there is a desperate need for new treatment strategies (Burnette et al., 2022). Some have shown very promising results, while others have not proven useful. Some others, such as GHB, have shown promising results but due to a high abuse risk seem ill-suited to patients with a substance use disorder. Other medications, such mifepristone and again GHB, pose a risk of criminality, in the case of mifepristone, illegal abortions, and in the case of GHB, drug-facilitated sexual assault. New treatment strategies such as controlled drinking, which overturns the widespread dogma of abstinence, also deserve to be considered (Simpson et al., 2018; Henssler et al., 2021; Paquette et al., 2022). To provide a convenient overview of off-label medications currently under investigation, the results above are summarized in a Table 1 below. We have also provided a rating of existing scientific data, ranking the data on medications as “good,” “intermediate,” “limited” or “poor” in descending order, as well as noting “only preclinical trials” in cases where scientific data does not include human clinical trials. We also evaluated results with regard to their success in treating alcohol use disorder, rating outcomes from “very promising,” “promising,” “conflicting,” “disappointing” to “very disappointing.” In case where no conclusion could be drawn, the results were labeled “inconclusive.” Furthermore, we provide recommendations for further scientific studies where we either recommend conducting further preclinical or clinical studies or suggest abandoning a therapeutic approach if data disappoint. Some well-established medications, supported by good data, have been recommended as possible second-line or additional therapies. Naturally, the implementation of potential therapeutic standards will require careful monitoring in broad clinical studies (Tanner et al., 2022).

**TABLE 1:** Off-label and investigational drugs in the treatment of alcohol use disorder: overview.

Medication	Results	Data	Recommendation	Mechanism of action	Medication group	Advantage	Disadvantage
**Baclofen**	Conflicting (promising, but serious safety concerns)	Good	Second-line therapy	GABA-B agonist	Muscle relaxant	Renal excretion	Dosing 3–4x/day
							Overdose risk with severe sedation and coma
**Gabapentin**	Conflicting (promising)	Good	Additional therapy	Inhibition of Na+ and Ca2+ cannels	Anticonvulsant	Reduction of heavy drinking	Overdose risk
			Reduction of heavy drinking				
**Topiramate**	Promising	Intermediate	Additional therapy	GABAergic inhibition	Anticonvulsant	Mainly renal excretion	Moderate effect
			Second-line therapy				
**Ondansetron**	Highly promising	Good	Combined therapy	Serotonin antagonist	Antiemetic	Especially for early onset alcoholism	Pharmacogenetic differences
**Varenicline**	Highly promising	Good	Combined therapy, especially for comorbid nicotine abuse	nAChR agonist	Smoking cessation	Nearly no hepatic metabolism	Not for maintaining abstinence, more for drinking reduction
**Aripiprazole**	Conflicting (promising)	Good	Second-line therapy for high-impulsivity and low self-control	Dopamine receptor agonist, serotonin receptor agonist and antagonist	Antipsychotic	For high-impulsivity	Pharmacogenetic differences
							Adverse events
**Quetiapine**	Highly disappointing	Intermediate	Not useful for treatment of alcohol use disorder	Serotonin and dopamine antagonist	Antipsychotic	Craving reduction only in patients with insomnia	Risk of misuse
**Clozapine**	Disappointing	Poor	Not useful for treatment of alcohol use disorder	Multiple neurotransmission pathways	Antipsychotic	Limited evidence for craving reduction	Risk of misuse
**Antidepressants**	Disappointing (except SSRI)	Limited	Further preclinical and clinical testing	Multiple mechanism of action depending on the drug	Antidepressants	Small reduction of alcohol consumption with SSRI.	Risk of misuse
							Possible pharmacogenetic differences
**Lithium**	Highly disappointing	Limited	Not useful for treatment of alcohol use disorder	Unknown mechanism of action	Mood stabilizer	No benefits for alcohol use disorder	Adverse events
**Neuropeptide Y**	Promising	Only preclinical studies	Further preclinical and clinical testing	G-protein coupled Y1-Y6 receptors	Neuropeptide	Preventing progression to addiction	Possible pharmacogenetic differences
**Neuropeptide S**	Conflicting (promising)	Only preclinical studies	Further preclinical and clinical testing	G-protein coupled neuropeptide S receptor	Neuropeptide	Anxiolytic effect	Possible diverse pharmacogenetic differences
**CRF antagonists**	Conflicting (promising)	Poor	Further preclinical and clinical testing and development of slow-offset antagonists	CRF1 and CRF2 receptors	Neuropeptide	Promising effect in preclinical studies	Poor translation from rodent studies to clinical human studies
**Oxytocin**	Highly promising (in rodent studies)	Poor	Further preclinical and clinical testing	Inhibition of growth hormone-releasing factor interactions with GABAergic interneurons in amygdala	Neuropeptide	Very good safety profile and mainly positive side effects	Very short half-life of intranasal oxytocin
			Perhaps as needed use				Poor translation from rodents to humans
**PF-05190457**	Highly promising	Poor	Further preclinical and clinical testing	Ghrelin receptor inverse agonist	Gastrointestinal peptide hormone system	Good safety profile	Dosage 3–4x per day
**Memantine**	Disappointing	Limited	Not useful as an anti-craving drug in treatment of alcohol use disorder	Non-competitive NMDA receptor antagonist	Treatment of Alzheimer disease	Maybe useful for alcohol-cue stimuli deconditioning	No effect on craving
**Ifenprodil**	Promising	Poor	Further preclinical and clinical testing	Non-competitive NMDA receptor antagonist	NMDA receptor modulator	Reduction of alcohol consumption in preclinical and clinical trials	Very limited data
**Samidorphan**	Promising	Poor	Further preclinical and clinical testing	μ-opioid receptor antagonist	Opioid system modulator	Favorable side effect profile	Somnolence and small risk of abuse
**Ondelopran**	Promising	Poor	Further preclinical and clinical testing	Selective opioid receptor antagonist	Opioid system modulator	Reduction of alcohol consumption	Very high interindividual pharmacokinetic variability
**ABT-436**	Conflicting (disappointing)	Poor	Probably not useful for treatment of alcohol use disorder	V1B antagonist	Vasopressin receptor antagonist	Favorable results for patients with high stress levels	Conflicting results within the study
**SSR149415**	Promising	Only preclinical studies	Further preclinical and clinical studies	V1B antagonist	Vasopressin receptor antagonist	Synergetic effect with naltrexone	Very limited preclinical data
**Mifepristone**	Conflicting (promising, but with very high misuse, risk of illegal abortion)	Limited	Not recommended in treatment of alcohol use disorder	Progesterone and type II glucocorticoid receptor antagonist	Medical abortion of pregnancy	Reduction of alcohol-induced neuro-degeneration	Very high risk of misuse for illegal abortion
**Ibudilast**	Conflicting (disappointing)	Poor	Further clinical testing	Non-selective phosphodiesterase inhibitor	Anti-inflammatory and treatment of asthma	Reduction of craving with depressive comorbidity	No craving reduction in general alcohol use disorder
**Citicoline**	Highly disappointing	Poor	Not useful for treatment of alcohol use disorder	Cell-membrane phospholipid intermediate product	Neuronal cell-membrane modulator	Reduction of cocaine consumption but with habituation effect	No effect at all on alcohol consumption and craving
**Rimonabant**	Highly disappointing	Poor	Not useful for treatment of alcohol use disorder	Selective CB1 receptor blocker	Endocannabinoid-system modulator	Treatment of tobacco smoking, obesity and cardiometabolic risk factors	Depression-related adverse events, anxiety and suicidal tendencies
**Surinabant**	Inconclusive	Only preclinical studies	Not useful for treatment of alcohol use disorder	CB1 receptor antagonist	Endocannabinoid-system modulator	Reduction of alcohol consumption in rats	No clinical data due to severe adverse events of rimonabant
**AM4113**	Promising	Only preclinical studies	Further preclinical and clinical testing	CB1 receptor neutral antagonist	Endocannabinoid-system modulator	In preclinical tests no anxiety and depression-like effects	No clinical data
**Gamma-hydroxybutyrate (GHB)**	Highly promising (but high risk of abuse)	Good	Not recommended by WHO for treatment of alcohol use disorder due to high abuse risk	GHB receptor and partial GABA-B receptor agonist	Treatment of narcolepsy and cataplexy	Very promising results for reduction of alcohol consumption	High abuse risk and misuse as rape drug. High risk of neuro-depression in case of co-ingestion with alcohol

For reasons of clarity, we do not cite sources for this table. These can all be found in the respective medication chapter in the text above. For further information, therefore please refer to the respective chapter and the cited literature.

**FIGURE 1:**
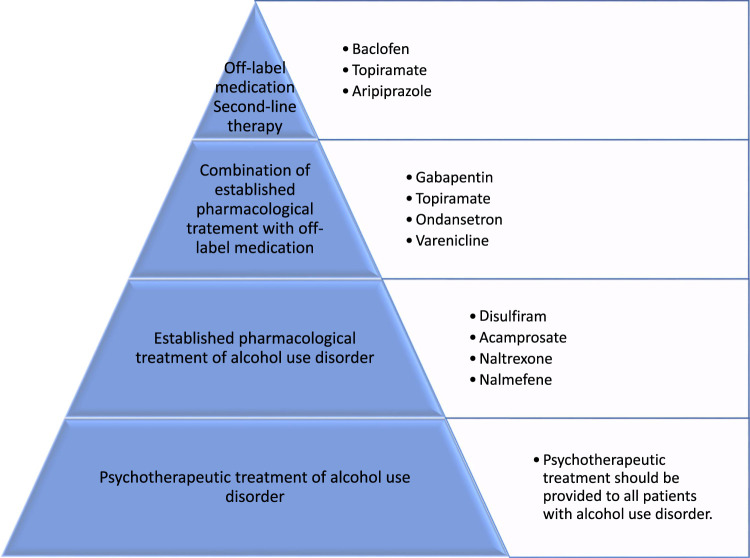
Pyramid of treatment in alcohol use disorder.

Taken together, the most promising results were found for topiramate, ondansetron, varenicline, neuropeptide Y, oxytocin, ghrelin inverse agonist and GHB. On the other hand, these medications achieved quite diverse ratings regarding scientific data. Some are quite well established, such as topiramate, ondansetron and varenicline, while others have only recently been investigated in the context of alcohol use disorder and data are therefore still very limited. Some medications may also be more useful for drinking reduction than for maintaining abstinence (Falk et al., 2019a), and beside abstinence treatment, harm-reduction strategies should also be taken into account (Witkiewitz et al., 2021). On the other hand, we also need to discuss the fact that all therapeutic strategies are limited to either targeting symptomatic improvement (suppression of alcohol-craving) or are disease-modifying (through classic deconditioning with the aversive agent disulfiram). To date, no treatment method has been shown to permanently cure the underlying neurobiological cause of alcohol use disorder (Figure 1). Therefore, all therapeutic strategies carry the risk of relapse, a risk that, due to altered neurotransmitter systems in the pathogenesis of alcohol use disorder, cannot be completely eliminated. As shown in our table overview, further preclinical or clinical studies are needed in order to precisely evaluate many of these medications, either as further scientific exploration or for implementation as second-line or additional therapeutic standards for the treatment of alcohol use disorder. Further research will generate new data concerning known medications and introduce new pharmacologic medications for use in substance use disorders. Moreover, progress in genetic knowledge and the implementation of pharmacogenetics in the allocation of patients to particular treatments will produce better therapeutic outcomes in alcohol use disorder. Some medications have already been identified as more effective in patient subgroups with a given genetic polymorphism.

In conclusion, we hope this review will aid researchers in their efforts to prioritize medications with promising results and to abandon those that have disappointed. In order to improve therapeutic outcomes many more preclinical and clinical studies of alcohol use disorder and relapse prophylaxis will undoubtedly be needed.
