# Supplementary figures and images for: Off-label and investigational drugs in the treatment of alcohol use disorder: A critical review
Source: Front Pharmacol. 2022 Oct 3;13:927703. doi: 10.3389/fphar.2022.927703 (PMC9574013; doi:10.3389/fphar.2022.927703)

# Pyramid of treatment in alcohol use disorder

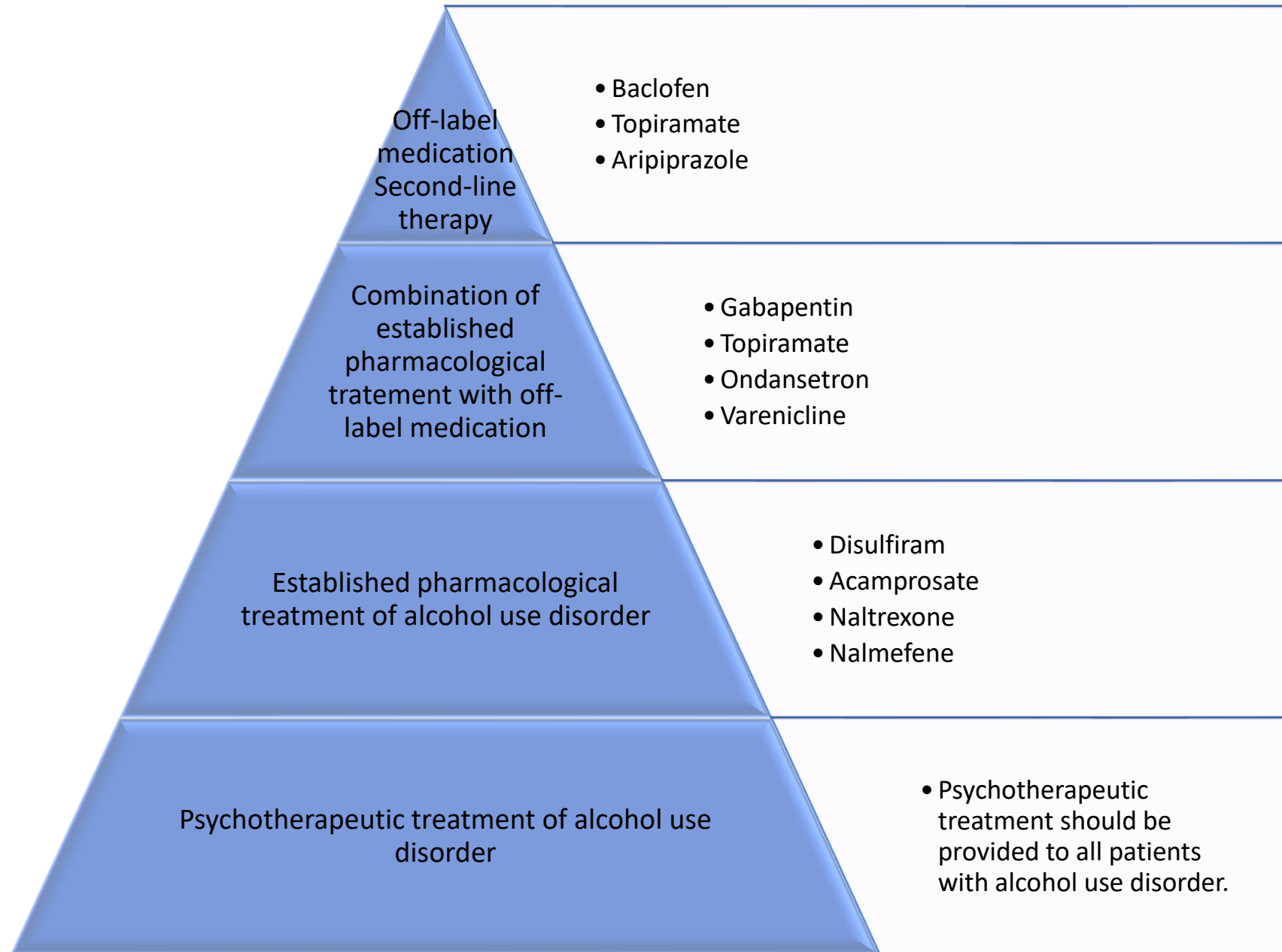

Supplement: Supplementary file 1 [file Image1.pdf]
